# Supplementary material for: Subgrouping and TargetEd Exercise pRogrammes for knee and hip OsteoArthritis (STEER OA): a systematic review update and individual participant data meta-analysis protocol
Source: BMJ Open. 2017 Dec 22;7(12):e018971. doi: 10.1136/bmjopen-2017-018971 (PMC5770908; doi:10.1136/bmjopen-2017-018971)
Supplement: Supplementary file 1 [file bmjopen-2017-018971supp001.pdf]

Appendix 1: Full search strategies for Medline and Embase

Medline search	Embase search
1. randomized controlled trial.pt.<br>2. controlled clinical trial.pt.<br>3. randomized.ab.<br>4. placebo.ab.<br>5. drug therapy.fs.<br>6. randomly.ab.<br>7. trial.ab.<br>8. groups.ab.<br>9. therapy.fs.<br>10. 1 or 2 or 3 or 4 or 5 or 6 or 7 or 8 or 9<br>11. limit 10 to humans<br>12. exp Exercise Movement Techniques/ or exp Exercise/ or exp Exercise Therapy/<br>13. ((strength$ or isometric$ or isotonic$ or isokinetic$ or endurance or weight$) adj5 train$).ti,ab.<br>14. (resistance adj5 (exercis$ or train$)).ti,ab.<br>15. ((physical$ or motion$) adj5 (fit$ or therap$)).ti,ab.<br>16. (physical$ adj5 activ$).ti,ab.<br>17. physiotherap$.ti,ab.<br>18. kinesiotherap$.ti,ab.<br>19. rehab$.mp.<br>20. exp Physical Fitness/<br>21. (walk$ or jog$ or run$ or cycl$ or swim$ or treadmill$ or gym$ or bicycl$ or skat$ or row$).ti,ab.<br>22. exp Sports/<br>23. sport$.ti,ab.<br>24. exercise$.ti,ab.<br>25. aerobic$.ti,ab.<br>26. yoga.mp. or (tai adj1 (chi or ji)).ti,ab. [mp=title, original title, abstract, name of substance word, subject heading word, unique identifier]<br>27. danc$.ti,ab.<br>28. (aqua$ or water).ti,ab.<br>29. hydro$.ti,ab.<br>30. exp Hydrotherapy/<br>31. (stretch$ or flexib$ or balanc$ or propriocept$).ti,ab.<br>32. (circuit$ adj1 train$).ti,ab.<br>36. 12 or 13 or 14 or 15 or 16 or 17 or 18 or 19 or 20 or 21 or 22 or 23 or 24 or 25 or 26 or 27 or 28 or 29 or 30 or 31 or 32<br>37. exp Osteoarthritis/<br>38. osteoarthr$.ti,ab.<br>39. OA.ti,ab.<br>40. degenerative arthritis.mp.<br>41. arthrosis.mp.<br>42. 37 or 38 or 40 or 41<br>43. exp Knee Joint/ or exp Knee/<br>44. exp Hip Joint/ or exp Hip/<br>45. (knee$ or hip$).ti,ab.<br>46. 43 or 44 or 45<br>47. 42 and 46<br>48. ((knee$ or hip$) adj5 pain).ti,ab.<br>49. 47 or 48<br>50. 11 and 36 and 49	1. crossover procedure/<br>2. double-blind procedure/<br>3. randomized controlled trial/<br>4. single-blind procedure/<br>5. random$.ti,ab.<br>6. factorial$.ti,ab.<br>7. crossover$.ti,ab.<br>8. cross over$.ti,ab.<br>9. placebo$.ti,ab.<br>10. (doubl$ adj blind$).ti,ab.<br>11. (singl$ adj blind$).ti,ab.<br>12. assign$.ti,ab.<br>13. allocat$.ti,ab.<br>14. volunteer$.ti,ab.<br>15. 1 or 2 or 3 or 4 or 5 or 6 or 7 or 8 or 9 or 10 or 11 or 12 or 13 or 14<br>16. exp exercise/<br>17. exp kinesiotherapy/<br>18. fitness/<br>19. ((strength$ or isometric$ or isotonic$ or isokinetic$ or endurance or weight$) adj5 train$).ti,ab.<br>20. (resistance adj5 (exercis$ or train$)).ti,ab.<br>21. ((physical$ or motion$) adj5 (fit$ or therap$)).ti,ab.<br>22. (physical$ adj5 activ$).ti,ab.<br>23. physiotherap$.ti,ab.<br>24. kinesiotherap$.ti,ab.<br>25. rehab$.mp.<br>26. (walk$ or jog$ or run$ or cycl$ or swim$ or treadmill$ or gym$ or bicycl$ or skat$ or row$).ti,ab.<br>27. sport$.ti,ab.<br>28. exp SPORT/<br>29. exercise$.ti,ab.<br>30. aerobic$.ti,ab.<br>31. (yoga or (tai adj1 (chi or ji))).ti,ab.<br>32. danc$.ti,ab.<br>33. (aqua$ or water).ti,ab.<br>34. hydro$.ti,ab.<br>35. exp hydrotherapy/<br>36. (stretch$ or flexib$ or balanc$ or propriocept$).ti,ab.<br>37. (circuit$ adj1 train$).ti,ab.<br>38. or/16–37<br>39. exp OSTEOARTHRITIS/<br>40. osteoarthr$.ti,ab.<br>41. OA.ti,ab.<br>42. degenerative arthritis.mp.<br>43. arthrosis.mp.<br>44. 39 or 40 or 41 or 42 or 43<br>45. exp knee/<br>46. exp HIP/<br>47. (knee$ or hip$).ti,ab.<br>48. 45 or 46 or 47<br>49. 44 and 48<br>50. ((knee$ or hip$) adj5 pain).ti,ab.<br>51. 49 or 50<br>52. 15 and 38 and 51<br>53. limit 52 to human
