# Supplementary material for: Subgrouping and TargetEd Exercise pRogrammes for knee and hip OsteoArthritis (STEER OA): a systematic review update and individual participant data meta-analysis protocol
Source: BMJ Open. 2017 Dec 22;7(12):e018971. doi: 10.1136/bmjopen-2017-018971 (PMC5770908; doi:10.1136/bmjopen-2017-018971)
Supplement: Author's manuscript [file bmjopen-2017-018971.draft_revisions.pdf]

BMJ Open

BMJ Open is committed to open peer review. As part of this commitment we make the peer review history of every article we publish publicly available.

When an article is published we post the peer reviewers' comments and the authors' responses online. We also post the versions of the paper that were used during peer review. These are the versions that the peer review comments apply to.

The versions of the paper that follow are the versions that were submitted during the peer review process. They are not the versions of record or the final published versions. They should not be cited or distributed as the published version of this manuscript.

BMJ Open is an open access journal and the full, final, typeset and author-corrected version of record of the manuscript is available on our site with no access controls, subscription charges or pay-per-view fees (http://bmjopen.bmj.com).

If you have any questions on BMJ Open's open peer review process please email editorial.bmjopen@bmj.com

BMJ Open

Subgrouping and TargetEd Exercise pRogrammes for knee and hip OsteoArthritis (STEER OA): A systematic review update and individual participant data meta-analysis protocol

Journal:	*BMJ Open*
Manuscript ID	bmjopen-2017-018971
Article Type:	Protocol
Date Submitted by the Author:	02-Aug-2017

Complete List of Authors:	Holden, Melanie; Keele University, Arthritis Research UK Primary Care Centre, Research Institute of Primary Care and Health Sciences<br>Burke, Danielle; Keele University, Arthritis Research UK Primary Care Centre, Research Institute of Primary Care and Health Sciences<br>Runhaar, Jos; Erasmus University Medical Center Rotterdam, Department of General Practice<br>van der Windt, Danielle; Keele University, Arthritis Research UK Primary Care Centre, Research Institute of Primary Care and Health Sciences<br>Riley, Richard; Keele University, Arthritis Research UK Primary Care Centre, Research Institute of Primary Care and Health Sciences<br>Dziedzic, Krysia; Keele University, Arthritis Research UK Primary Care Centre, Research Institute of Primary Care and Health Sciences<br>Legha, Amardeep; Keele University, Arthritis Research UK Primary Care Centre, Research Institute of Primary Care and Health Sciences<br>Evans, Amy; Keele University, Arthritis Research UK Primary Care Centre, Research Institute of Primary Care and Health Sciences<br>Abbott, J. Haxby; University of Otago, Centre for Musculoskeletal Outcomes Research, Orthopaedic Surgery Section, Department of Surgical Sciences, Dunedin School of Medicine<br>Baker, Kristin; Boston University, Sargent College<br>Brown, Jenny; Keele University, Research User Group, Arthritis Research UK Primary Care Centre, Research Institute of Primary Care and Health Sciences<br>Bennell, Kim; University of Melbourne, Centre for Health, Exercise & Sports Medicine, Department of Physiotherapy<br>Bossen, Daniël; Amsterdam University of Applied Sciences, ACHIEVE Centre of Expertise, Faculty of Health; University of Amsterdam, Coronel Institute of Occupational Health, Academic Medical Center<br>Brosseau, Lucie; University of Ottawa, Faculty of Health Sciences, School of Rehabilitation Sciences<br>Chaipinyo, Kanda; Srinakharinwirot University, Division of Physical Therapy, Faculty of Health Science<br>Christensen, Robin; Frederiksberg and Bispebjerg Hospital, Musculoskeletal Statistics Unit, The Parker Institute, Department of Rheumatology<br>Cochrane, Tom; University of Canberra, Centre for Research Action in Public Health<br>de Rooij, Mariette; Reade, Amsterdam Rehabilitation Research Centre, Centre for Rehabilitation and Rheumatology<br>Doherty, Michael ; University of Nottingham, Academic Rheumatology

	French, Helen; Royal College of Surgeons in Ireland, School of Physiotherapy<br>Hickson, Sheila; Keele University, Research User Group, Arthritis Research UK Primary Care Centre, Research Institute of Primary Care and Health Sciences<br>Hinman, Rana S.; University of Melbourne, Centre for Health, Exercise & Sports Medicine, Department of Physiotherapy<br>Hopman-Rock, M; TNO Netherlands Organisation for Applied Scientific Research; VU University Medical Center, The Netherlands Department of Public and Occupational Health, EMGO Institute for Health and Care Research<br>Hurley, Mike; St George's University of London and Kingston University, Faculty of Health, Social Care and Education; Health Innovation Network South London<br>Ingram, Carol; Keele University, Research User Group, Arthritis Research UK Primary Care Centre, Research Institute of Primary Care and Health Sciences<br>Knoop, J; Reade, Amsterdam Rehabilitation Research Centre, Centre for Rehabilitation and Rheumatology<br>Krauss, Inga; University Hospital of Tübingen, Department of Sports Medicine, Medical Clinic<br>McCarthy, Chris; Manchester Metropolitan University, Manchester Movement Unit, Manchester School of Physiotherapy<br>Messier, Stephen; Wake Forest University, J.B. Snow Biomechanics Laboratory, Department of Health and Exercise Science, Worrell Professional Center<br>Patrick, Donald; University of Washington, Department of Health Services<br>Sahin, Nilay; Balikesir Universitesi, Department of Physical Medicine and Rehabilitation, Medical Faculty<br>Talbot, Laura; University of Tennessee Health Science Center, College of Medicine, Department of Neurology<br>Taylor, Robert; Keele University, Research User Group, Arthritis Research UK Primary Care Centre, Research Institute of Primary Care and Health Sciences<br>Teirlinck, Carolien; Erasmus University Medical Center Rotterdam, Department of General Practice<br>van Middelkoop, Marienke; Erasmus University Medical Center Rotterdam, Department of General Practice<br>Walker, Christine; Keele University, Research User Group, Arthritis Research UK Primary Care Centre, Research Institute of Primary Care and Health Sciences<br>Foster, Nadine; Keele University, Arthritis Research UK Primary Care Centre, Research Institute of Primary Care and Health Sciences
**Primary Subject Heading**:	Rheumatology
Secondary Subject Heading:	Rehabilitation medicine
Keywords:	Musculoskeletal disorders < ORTHOPAEDIC & TRAUMA SURGERY, Hip < ORTHOPAEDIC & TRAUMA SURGERY, Knee < ORTHOPAEDIC & TRAUMA SURGERY, osteoarthritis, exercise

SCHOLARONE™
Manuscripts

TITLE: Subgrouping and TargetEd Exercise pRogrammes for knee and hip OsteoArthritis (STEER OA): A systematic review update and individual participant data meta-analysis protocol

AUTHORS:

1. Melanie A Holden[1] m.holden@keele.ac.uk
2. Danielle L Burke[1] d.burke@keele.ac.uk
3. Jos Runhaar[2] j.runhaar@erasmusmc.nl
4. Danielle van Der Windt[1] d.van.der.windt@keele.ac.uk
5. Richard D Riley[1] r.riley@keele.ac.uk
6. Krysia Dziedzic[1] k.s.dziedzic@keele.ac.uk
7. Amardeep Legha[1] a.legha@keele.ac.uk
8. Amy L Evans[1] a.evans1@keele.ac.uk
9. Haxby Abbott[3] haxby.abbott@otago.ac.nz
10. Kristin Baker[4] krbaker@bu.edu
11. Jenny Brown[5]
12. Kim L Bennell[6] k.bennell@unimelb.edu.au
13. Daniël Bossen[7,8] d.bossen@hva.nl
14. Lucie Brosseau[9] lbrossea@uottawa.ca
15. Kanda Chaipinyo[10] kanda@g.swu.ac.th
16. Robin Christensen[11] robin.christensen@regionh.dk
17. Tom Cochrane[12] Tom.Cochrane@canberra.edu.au
18. Mariette de Rooij[13] m.d.rooij@reade.nl
19. Michael Doherty[14] Michael.Doherty@nottingham.ac.uk
20. Helen P French[15] hfrench@rcsi.ie

21. Sheila Hickson[5]

22. Rana S Hinman[6] ranash@unimelb.edu.au

23. Marijke Hopman-Rock[16,17] m.hopman@vumc.nl

24. Michael V Hurley[18,19] Michael.Hurley@sgul.kingston.ac.uk

25. Carol Ingram[5]

26. Jesper Knoop[13] j.knoop@reade.nl

27. Inga Krauß[20] Inga.Krauss@med.uni-tuebingen.de

28. Chris McCarthy[21] cmccarthy@mmu.ac.uk

29. Stephen P Messier[22] messier@wfu.edu

30. Donald L. Patrick[23] donald@uw.edu

31. Nilay Sahin[24] nilaysahin@gmail.com

32. Laura A Talbot[25] ltalbot@uthsc.edu

33. Robert Taylor[5]

34. Carolien H Teirlinck[2] c.teirlinck@erasmusmc.nl

35. Marienke van Middelkoop[2] m.vanmiddelkoop@erasmusmc.nl

36. Christine Walker[5]

37. Nadine E Foster[1] n.foster@keele.ac.uk

In collaboration with the OA Trial Bank

Affiliations:

1. Arthritis Research UK Primary Care Centre, Research Institute of Primary Care and Health Sciences, Keele University, Keele, United Kingdom.

2. Erasmus University Medical Center Rotterdam, Department of General Practice, Rotterdam, the Netherlands.

For peer review only

3. Centre for Musculoskeletal Outcomes Research, Orthopaedic Surgery Section, Department of Surgical Sciences, Dunedin School of Medicine, University of Otago, Dunedin, New Zealand.

4. Sargent College, Boston University, Boston, United States of America.

5. Research User Group, Arthritis Research UK Primary Care Centre, Research Institute of Primary Care and Health Sciences, Keele University, Keele, United Kingdom.

6. Centre for Health, Exercise & Sports Medicine, Department of Physiotherapy, University of Melbourne, Melbourne, Australia.

7. ACHIEVE Centre of Expertise, Faculty of Health, Amsterdam University of Applied Sciences, Amsterdam, the Netherlands.

8. Coronel Institute of Occupational Health, Academic Medical Center, University of Amsterdam, Amsterdam, the Netherlands.

9. Faculty of Health Sciences, School of Rehabilitation Sciences, University of Ottawa, Ottawa, Canada.

10. Division of Physical Therapy, Faculty of Health Science, Srinakharinwirot University, Bangkok, Thailand.

11. Musculoskeletal Statistics Unit, The Parker Institute, Department of Rheumatology, Frederiksberg and Bispebjerg Hospital, Denmark.

12. Centre for Research & Action in Public Health, University of Canberra, Canberra, Australia.

13. Amsterdam Rehabilitation Research Center, Reade, Centre for Rehabilitation and Rheumatology, Amsterdam, the Netherlands.

14. Academic Rheumatology, University of Nottingham, Nottingham, United Kingdom

15. School of Physiotherapy, Royal College of Surgeons in Ireland, Dublin, Ireland.

For peer review only

16. TNO Netherlands Organisation for Applied Scientific Research, Leiden, the Netherlands.

17. The Netherlands Department of Public and Occupational Health, EMGO Institute for Health and Care Research, VU University Medical Center, Amsterdam, the Netherlands.

18. Faculty of Health, Social Care and Education, St George's University of London and Kingston University, London, United Kingdom.

19. Health Innovation Network South London, London, United Kingdom.

20. Department of Sports Medicine, Medical Clinic, University Hospital of Tübingen, Tübingen, Germany.

21. Manchester Movement Unit, Manchester School of Physiotherapy, Manchester Metropolitan University, United Kingdom.

22. J.B. Snow Biomechanics Laboratory, Department of Health and Exercise Science, Worrell Professional Center, Wake Forest University, Winston Salem, United States of America.

23. Department of Health Services, University of Washington, Washington, United States of America.

24. Department of Physical Medicine and Rehabilitation, Medical Faculty, Balıkesir University, Balıkesir, Turkey

25. University of Tennessee Health Science Center, College of Medicine, Department of Neurology, Memphis, United States of America.

Corresponding author: Dr Melanie Holden, Arthritis Research UK Primary Care Centre, Research Institute of Primary Care and Health Sciences, Keele University,

For peer review only

Keele, Staffordshire, ST5 5BG. Email: m.holden@keele.ac.uk, Telephone: (+44) 01782 734720

WORD COUNT: 3951

ABSTRACT

Introduction: Knee and hip osteoarthritis (OA) is a leading cause of disability worldwide. Therapeutic exercise is a recommended core treatment for people with knee and hip OA, however, the observed effect sizes for reducing pain and improving physical function are small to moderate. This may be due to insufficient targeting of exercise to subgroups of people who are most likely to respond, and/or sub-optimal content of exercise programmes. This study aims to identify a) subgroups of people with knee and hip OA that do/ do not respond to therapeutic exercise, and to different types of exercise, and b) mediators of the effect of therapeutic exercise for reducing pain and improving physical function. This will enable optimal targeting and refining the content of future exercise interventions.

Methods and analysis: Systematic review and individual participant data meta-analyses. A previous comprehensive systematic review will be updated to identify randomised controlled trials that compare the effects of therapeutic exercise for people with knee and hip OA on pain and physical function, to a non-exercise control. Lead authors of eligible trials will be invited to share individual participant data. Trial- and participant-level characteristics (for baseline variables and outcomes) of included studies will be summarised. Meta-analyses will use a two-stage approach, where effect estimates are obtained for each trial and then synthesised using a random effects model (to account for heterogeneity). All

For peer review only

analyses will be on an intention-to-treat principle and all summary meta-analysis estimates will be reported as standardised mean differences with 95% confidence intervals. **Ethics and dissemination:** Research ethical or governance approval is exempt as no new data are being collected and no identifiable participant information will be shared. Findings will be disseminated via national and international conferences, publication in peer-reviewed journals, and summaries posted on websites accessed by the public and clinicians. **REGISTRATION DETAILS:** PROSPERO: CRD42017054049

STRENGTHS AND LIMITATIONS OF THIS STUDY

1. This will be the first study in the field of therapeutic exercise and osteoarthritis to combine individual participant data from existing randomised controlled trials.
2. Combining individual participant data from existing trials will increase the power to identify who benefits most from therapeutic exercise, and to identify underlying mechanisms of action.
3. Individual participant data meta-analyses facilitates standardised analyses across studies, allows direct derivation of desired information independent of significance or reporting, enables subgroup effects and interactions (differences in effects between subgroups) to be examined, and may provide more outcomes than were considered in a single original publication.
4. A disadvantage to completing individual participant data meta-analyses is the time required to complete them, including obtaining, checking, and combining the data.

For peer review only

INTRODUCTION

Osteoarthritis (OA) can be defined as a clinical syndrome of joint pain accompanied by varying degrees of functional limitation and reduced quality of life[1]. OA, particularly of the knee and hip, is one of the leading causes of disability worldwide, with an estimated global age-standardised prevalence of 3.8% (95% confidence interval (CI) 3.6% to 4.1%) for knee OA and 0.85% (95% CI 0.74% to 1.02%) for hip OA[2]. The burden of OA will increase as the population ages and the prevalence of obesity rises[2,3].

No cure currently exists for OA and as such treatment strategies aim to reduce pain and improve physical function, and enhance quality of life[4]. Clinical guidelines, including the National Institute for Health and Care Excellence (NICE) OA guidelines[1], consistently recommend therapeutic exercise as a core treatment for people with knee and hip OA[5,6]. Therapeutic exercise involves participation in physical activity that is planned, structured, repetitive and purposeful for the improvement or maintenance of a specific health condition such as OA[7]. It encompasses general aerobic exercise, strengthening, flexibility, balance or body-region specific exercises[7]. Although both general (aerobic) exercise and strengthening exercise are recommended for people with knee and hip OA, limited information is available on the optimal prescription of therapeutic exercise (e.g. the optimal type, dose, intensity, and setting of exercise, and how best to progress exercise)[1]. Numerous systematic reviews and meta-analyses support the role of therapeutic exercise for knee and hip OA, consistently demonstrating that it can reduce pain and improve physical function[8-10]. However, results from randomised controlled trials (RCTs) show the observed effect sizes from exercise interventions

For peer review only

are small to moderate, can decline over time, and only approximately 50% of participants achieve a clinically important treatment response[11-13]. The modest average benefits obtained from therapeutic exercise could be due to the inclusion of subgroups of people who are unlikely to benefit from exercise, and thus the overall effect is closer to the null than if the trial had been solely undertaken in those that are likely to benefit[4]. Better targeting of exercise treatments for people with knee and hip OA could potentially lead to improved treatment effects and patient outcomes, as well as more efficient use of healthcare services, in a similar way as demonstrated for low back pain[14,15]. Such an approach requires the identification of subgroups who are likely to respond better to therapeutic exercise than others.

Little previous research has examined whether outcomes from exercise for OA vary for subgroups of people defined by individual-level characteristics (treatment effect moderators)[16]. Exploratory secondary analyses of some RCTs suggest a range of potential moderators of the effects of exercise, including age[17], sex[18], obesity[19], pain severity and duration[17,18], functional ability[18], strength[20,21], knee malalignment[19,20], severity of joint damage[22], anxiety and depression[18], and treatment outcome expectations[23]. However, post hoc analyses have low statistical power to detect significant subgroup effects, and are at high risk of yielding spurious findings due to multiple testing[24].Although these exploratory subgroup analyses are inconclusive, they add credence to the hypothesis that not all people with knee and hip OA respond similarly to exercise, with variability in effects related to individual-level characteristics.

For peer review only

Modest average benefits of therapeutic exercise in people with knee and hip OA may additionally be explained by sub-optimal content of exercise programmes. Systematic reviews have identified various characteristics of exercise programmes that appear to be associated with larger effects, but with conflicting results[10,25]. Treatment mediators (causal links between treatment and outcome[16]) of therapeutic exercise on OA symptoms are largely unknown, making it difficult to design therapeutic exercise programmes for optimal effects on symptoms. If true mediators were identified and targeted, the positive effects of therapeutic exercise may be improved. Increased muscle strength, decreased extension deficits and improved proprioception have been identified as potential working mechanisms for the positive effect of therapeutic exercise for knee OA, and increased muscle strength for hip OA[26]. However, meta-analyses at the study-level (using aggregated study results)[26] have been prone to study-level confounding regarding the identification of individual-level effects[25,26].

The investigation of individual response to exercise treatment, and the identification of factors that may cause differential response to such treatment or to particular components of exercise therapy, requires individual-level data analysis. To our knowledge, this type of trial data pooling and analysis has not yet been completed in the field of therapeutic exercise and OA. Although several systematic reviews are available[8-10], none use individual participant data (IPD). Given there are now over 60 RCTs of exercise for knee and hip OA[10] it is timely to combine IPD from these existing trials. This will increase the power to identify who benefits most from therapeutic exercise, and to identify underlying mechanisms of action [27]. IPD meta-analysis facilitates standardised analyses across studies, allows direct derivation of

For peer review only

desired information independent of significance or reporting, enables subgroup effects and interactions (differences in effects between subgroups) to be examined, and may provide longer follow-up, more participants, and more outcomes than were considered in the original publication[27]. Therefore IPD meta-analyses are potentially more reliable than traditional aggregate data meta-analyses for the identification of treatment effect moderators, may lead to different conclusions, and may produce more clinically relevant results[27].

AIM

To identify a) subgroups of people with knee and hip OA that respond/ do not respond to therapeutic exercise and to different types of exercise (effect moderators), and b) mediators of the effect of therapeutic exercise for reducing pain and improving physical function, to facilitate better targeting of future exercise interventions and refine exercise program content.

Specific analytic objectives

1. Determine the short- (12 weeks), medium- (6 months) and long-term (1 year) overall effects of therapeutic exercise on pain and physical function for people with knee and hip OA compared to a non-exercise control.
2. Determine which study-level characteristics of therapeutic exercise interventions are associated with improved overall effects on pain and physical function in people with knee and hip OA, including the type, intensity, duration, setting and deliverer of exercise.

For peer review only

3. Identify individual-level characteristics of people with knee and hip OA that are associated with the short-, medium-, and long-term effects of therapeutic exercise on pain and physical function.
4. Identify individual-level characteristics of people with knee and hip OA that are associated with the effects of different characteristics of therapeutic exercise interventions; including the type, intensity, duration, setting, and deliverer of exercise.
5. Investigate the effect estimates for objectives 1 to 4 in subgroups of people with only knee OA and only hip OA to examine whether they differ by joint site.
6. Evaluate the mediating effects of muscle strength (for people with knee and hip OA), proprioception (for people with knee OA) and extension deficits (for people with knee OA) in the association between therapeutic exercise and pain and physical function. The effects of individual and combined mediators will be explored.

METHODS AND ANALYSIS

We will update a previous systematic review[10] to identify relevant RCTs and after agreement from trial leads, undertake an IPD meta-analysis. Our systematic review and meta-analysis will be completed in accordance with methods advocated by the Cochrane IPD meta-analysis group[28,29], and reported according to PRISMA-IPD guidance[30]. Five members of the Research User Group at the Research Institute of Primary Care and Health Sciences, Keele University, have formed a Patient and Public Involvement and Engagement (PPIE) working group for this study. In line with INVOLVE[31] there will be PPIE involvement at every stage of the project. We will work in collaboration with the OA Trial Bank (www.oatrialbank.com), an initiative

For peer review only

established in 2010 to collect and analyse IPD of published RCTs in OA[32,33]. The final IPD database will be deposited with the OA Trial Bank for the benefit of the wider OA research community.

PHASE 1: TRIAL IDENTIFICATION

We previously conducted a systematic review that identified 60 RCTs of exercise interventions for people with knee and hip OA that are relevant for inclusion within this study[10]. We will update this review. The search strategy previously developed will be re-run from the date of the previous search (March 2012) in the following electronic databases: Medline, Embase, CINAHL, AMED, HMIC (Health Management Information Consortium), Cochrane Database of Systematic Reviews, Cochrane Controlled Clinical Trials (CENTRAL), DARE (Database of Reviews of Effectiveness), NHS EED (Economic Evaluations Database), and Web of Science. Bibliographies of relevant review articles and included articles will be examined for additional potentially relevant trials. There will be no language restriction. Full search strategies for Medline and Embase are shown in Appendix 1.

Study Selection

We will evaluate RCTs against the following inclusion criteria:

Study population: Adults with knee or hip OA; ***Intervention:*** Any land or water based therapeutic exercise intervention regardless of content, duration, frequency, or intensity; ***Comparator:*** Other forms of exercise or no exercise control group (including usual care, waiting list, placebo, attention control, or no treatment); ***Outcome measure:*** At least one measure of self-reported pain or physical function;

For peer review only

Study design: RCTs. Trials will be excluded if they concern pre- or post-operative therapeutic exercise, when exercise is combined with interventions other than advice/ education/ self-management/ motivational techniques (meaning treatment effects cannot clearly be attributed to the exercise), or if intervention groups receive identical therapeutic exercise interventions (Table 1). Titles and abstracts of identified studies and subsequently full papers will be independently reviewed by two reviewers. A third reviewer will be consulted to resolve disagreements if necessary.

Table 1: Inclusion/ exclusion criteria

	Inclusion criteria	Exclusion criteria
**Population**	• Knee and/or hip pain in adults aged 45 years and over (mean age over 45 years)<br>• Knee and/ or hip OA diagnosed by x-ray<br>• Knee and/ or hip OA diagnosed according to clinical criteria<br>• Knee and/ or hip OA diagnosed by health care professional<br>• Self-reported knee and/ or hip OA<br>N.B: If population is mixed (e.g. OA and rheumatoid arthritis (RA), include if over 50% of participants have OA	• Knee and/ or hip pain attributable to conditions other than OA<br>• Non-musculoskeletal conditions<br>• Rheumatoid arthritis/ other defined inflammatory rheumatological problems<br>• Pre-operative patients (people on waiting lists for knee/hip surgery, including total joint replacement)<br>• Post-operative patients (immediately following knee/hip surgery, including total joint replacement)<br>• People with 'patellofemoral pain syndrome' (overall a different problem to 'OA')<br>• Animal based studies<br>• Studies of children
**Interventio**	• Any therapeutic exercise	• Non exercise interventions

For peer review only

n	intervention (land or water based), regardless of content, duration, frequency, or intensity	• Advice only to exercise or increase physical activity, including within wider OA self-management programmes<br>• Exercise or physical activity that was not specifically applied to improve or maintain knee and/ or hip OA<br>• Exercise combined with other treatment modalities other than advice/ education/ self-management/ motivational techniques)<br>• Pre/post-operative exercise therapy, i.e. exercise immediately before, or following knee/hip surgery
**Comparator**	• Other forms of exercise (i.e. different type, duration, frequency or intensity of exercise if sufficiently different from the intervention arm), or no exercise control group (including usual care, waiting list, placebo, attention control, or no treatment)	• If intervention groups receive identical therapeutic exercise interventions (i.e. no contrast existing between the intervention groups)<br>• If the comparator is a different active intervention other than usual care, waiting list, placebo, attention control, or no treatment (for example intra-articular injection, opioids, weight loss etc.)
**Outcome measure**	• Any self-reported measure of pain and/or physical function	• No measure of self-reported pain and physical function
**Study design**	• Randomised controlled trial<br>• Quasi-randomised controlled trial (where the method of allocation is known, but is not considered strictly random, e.g. alternation,	• Non-randomised controlled trial study design<br>• Other study designs e.g. surveys, observational studies, pre-post experiments (without a control group), qualitative studies

For peer review only

	date of birth, medical record number).	• Systematic reviews<br>• RCT protocols

Extraction of aggregate data

For each included trial, details on design, sample size, population characteristics (knee OA, hip OA or mixed), interventions (type, duration, exercise deliverer), comparator, candidate baseline variables (potential treatment moderators and mediators), and outcome assessment (type of outcome measure, length of follow-up) will be extracted and summarised into tables. Two reviewers will independently extract outcome data on self-report pain and/or physical function at time points nearest to 12 weeks, 6 months and 12 months. Two reviewers will independently classify the exercise interventions of included trials, based on the following a priori defined characteristics:

Frequency of exercise: Number of exercise sessions per week.

Intensity of exercise: Low, moderate or high intensity; low, moderate or high impact (based on published information e.g. Metabolic Equivalent, one repetition maximum).

Type of exercise: Predominantly strengthening (e.g. quadriceps strengthening); predominantly general (aerobic) (e.g. walking, swimming); predominantly mind-body (e.g. yoga, tai-chi)[34]; mixed. As many trials are likely to include predominantly strengthening, we will classify subsets of predominantly non-weightbearing/ open kinetic chain strengthening exercise versus predominantly weightbearing/ closed kinetic chain strengthening exercise.

Duration of exercise programme: Short (less than 6 weeks), or longer durations of up to 12 weeks, and over 12 weeks; total number of exercise sessions; booster sessions or no booster sessions.

For peer review only

Setting of exercise: Group, individual or mixed; supervised in clinic, completed at home or mixed; face-to-face, remote exercise instruction, or mixed.

Exercise deliverer: Healthcare professionals, exercise specialists, peer or lay-led, or mixed.

PHASE 2: COLLECTION, CHECKING AND STANDARDISING INDIVIDUAL PARTICIPANT DATA

In collaboration with, and following the procedures of, the OA Trial Bank, we will contact lead authors of identified trials to inform them about the study and invite them to share IPD. If the updated systematic review yields a large additional number of RCTs suitable for inclusion, and many authors are willing to share IPD, we may examine the likely power of the IPD meta-analysis accordingly to the trials promising their IPD using a simulation-based approach[35]. The collection, cleaning and harmonisation of IPD is often resource intensive[28,36], and therefore the power calculation will inform how much IPD is required in order to obtain sufficient power (e.g. 80%) to evaluate our key objectives. If IPD is ultimately sought from a subset of studies, this will be based on study quality, sample size and improvement to power, and will be independent of effect size to avoid selection bias[37].

Once a data sharing agreement is in situ, datasets will be accepted in any form, provided all data are anonymised and variables and categories are adequately labelled in English. To ensure accurate pooling of data, each dataset will be converted to a common format and variables re-named in a consistent manner.

For peer review only

Variables of interest

IPD to be obtained (where available) will include the following:

Baseline measures:

Socio-demographic factors: age, sex, co-morbidities, frailty, fatigue/ vitality, quality of life, body mass index, baseline physical activity level, previous lower limb injury, work status (working yes/no), manual versus non-manual work, previous physical load, family history of OA, socioeconomic status (education), social support, currently receiving other treatment, smoking status, motivation to exercise, previous knee injury/ trauma.

Psychological factors: anxiety/ depression, self-efficacy, outcome expectations.

Disease characteristics: pain location, pain elsewhere, pain severity, pain duration, central pain sensitisation, pain bothersomeness, physical function, stage of OA (early versus established OA[38]), radiographic joint structure, evidence of synovitis and bone marrow lesions from MRIs, patellofemoral OA damage.

Biomechanical factors: proprioception, static/ dynamic alignment, strength of hip and lower limb musculature, range of movement, leg length discrepancy, developmental hip abnormalities/ deformities.

Outcome measures: All self-report pain and physical function outcome data at time-points nearest to 12 weeks (short-term), 6 months (mid-term) and 1 year (long-term). If more than one measure of self-reported pain and physical function are reported, we will chose the highest in the hierarchy of outcome measures, as recommended by the Cochrane Musculoskeletal Review Group[39]. For pain these are: 1. pain overall; 2. pain on walking; 3. WOMAC pain subscale; 4. pain on activities other than

For peer review only

walking; 5. WOMAC global scale; 6. Lequesne osteoarthritis index global score; 7. other algofunctional scale; 8. patient's global assessment; 9. physician's global assessment; 10. other outcome; 11. no continuous outcome reported. For physical function these are: 1. global disability score; 2. walking disability; 3. WOMAC disability subscore; 4. composite disability scores other than WOMAC; 5. disability other than walking; 6. WOMAC global scale; 7. Lequesne osteoarthritis index global score; 8. other algofunctional scale. Additionally, strength of hip and lower limb musculature, range of movement (total ROM, maximal flexion, maximal extension, extension deficit), and proprioception measures will be obtained during, and directly post-intervention.

Data quality assurance

We will evaluate the IPD from each trial to ensure the ranges of included variables are reasonable, and missing data will be checked against the original trial report. We will attempt to re-produce the results included in each initial trial publication, including baseline characteristics and self-reported pain and physical function at a time point nearest to 12 weeks, 6 months and 1 year. Discrepancies or missing information will be discussed and clarified with original trial authors. Where discrepancies cannot be explained the trial data will be excluded from the analysis. Individual trial datasets will be combined to form a new master dataset with a variable added to indicate the original trial.

Assessment of risk of bias

For peer review only

We will use the Cochrane Collaboration's tool for assessing risk of bias, based on publications of the included trials[40]. Two researchers will independently grade risk of bias (unclear, high, or low risk of bias) based on sequence generation, allocation concealment, blinding of outcome assessor, incomplete outcome data, and selective outcome reporting. Trial design, conduct and analysis methods will be clarified with Principal Investigators. Additionally, IPD will be directly checked for key potential biases, including whether baseline participant characteristics are balanced by arm. It will also be checked to ensure that data on all or as many randomised participants as possible are included, and any additional relevant outcome data from trials will be obtained.

PART 3: STATISTICAL ANALYSES

We will describe trial-level and participant-level characteristics (for baseline variables and outcomes) of included studies and examine if RCTs for which IPD are obtained are representative of the full set of existing RCTs by comparing key study characteristics, e.g. country of origin, sample size. All meta-analyses, apart from the mediation analyses (Objective 6 below), will use a two-stage approach, where each trial is analysed separately in the first stage (which accounts for clustering of participants within trials) to produce effect estimates of interest, which are then synthesised in the second stage to produce summary meta-analysis results based on a random effects model (to account for between-trial heterogeneity)[26]. Analyses will be on an intention-to-treat principle and all summary estimates will be reported with 95% confidence intervals and *p*-values, with approaches such as Hartung-Knapp used to account for uncertainty of variance estimates[41,42]. Under a

For peer review only

'missing-at-random' assumption, individuals with partially missing outcome data will be included in analyses without imputation using a longitudinal data meta-analysis framework. If there is a considerable amount of missing baseline data for particular variables of interest (such as potential individual-level effect moderators) this will be handled using within-study multiple imputation[43] and Rubin's rule to estimate effects from imputed datasets[44]. All analyses will be carried out using Stata 14.1[45] or SAS 9.3[46].

Objective 1: For the meta-analysis to estimate an overall intervention effect (at 12 weeks, 6 months and 1 year) for self-reported pain and physical function, all available comparisons will be grouped into any exercise intervention versus a non-exercise control. Most outcomes will be continuous, so linear regression models will be fitted in the first stage. Longitudinal models will be used to account for the correlation between outcome values at the multiple time-points[47]. For each trial, the model will include baseline pain/physical function, treatment, time, and treatment by time interaction terms. The second stage requires a multivariate meta-analysis framework, which jointly syntheses the treatment effect estimates from the multiple time-points across trials[48]. Given the likely heterogeneity in the intervention effects across trials (e.g. due to variability in patient characteristics), we will assume a multivariate random-effects meta-analysis model to estimate the summary results of interest using restricted maximum likelihood (REML) estimation. Heterogeneity in treatment effects across trials will be summarised by the estimated between-trial variance (τ^2) and multivariate I-squared statistics.

Objective 2: To determine which characteristics of exercise are associated with differences in overall effects, the meta-analysis approach in objective 1 will be repeated

for particular subgroups of exercise interventions, including types, intensities, duration, setting or deliverer of exercise. To formally evaluate (albeit indirect) differences between exercise subgroups, meta-regression will be used. Trials comparing two different forms of exercise interventions will also be summarised, as these give direct (within-trial) information, which is preferable to indirect information. As appropriate, a sensitivity analysis will be performed to include direct and indirect evidence in one large (network) meta-analysis model.

Objectives 3 and 4: The IPD will be further analysed to examine treatment effect modification at the patient level, where individual patient characteristics are associated with differences in response to exercise. The models fitted in each trial will additionally include interaction terms between the intervention and patient-level covariates of interest to test effect modification. The interaction effect estimates at each time-point will then be pooled across trials using a multivariate random-effects meta-analysis. Covariates will be mean centred to aid the translation of results. Analyses of different exercise interventions (objective 4) will also be extended to examine treatment-covariate interactions in the same way, and identify subgroups of individuals likely to benefit most from specific types, intensities, duration, setting, and deliverer of exercise.

Objective 5: The analyses described for objectives 1 to 4 will be repeated in subgroups of people with only knee OA and only hip OA to examine whether the effect estimates differ by joint site.

Objective 6: As depicted in Figure 1 (panel A), in a mediation analysis an exposure can affect the outcome either through the mediator (E→M→O) or through other pathways (E→O). Using the counterfactual approach[49], the *total effect* of the

For peer review only

exposure (exercise therapy) on the outcome (pain/physical function) through both pathways is determined. This effect can be decomposed into two components: the *direct effect* and the *indirect effect*. The *direct effect* refers to the causal pathway by which exercise therapy has an effect on pain/physical function not through the mediator. The *indirect effect* refers to the effect of exercise therapy that operates solely through the mediator under investigation. The counterfactual approach also allows for multiple mediators (Figure 1 panel B)[49]. Given the fact that in this approach the *total effect* can be decomposed into the *direct* and *indirect effects*, the percentage mediated by the mediator(s) can be calculated as an estimation of their importance.

In a one-stage approach, first the effect of the intervention (a) on the outcome (Y) will be determined, controlling for the mediator (m) under investigation and potential mediator-outcome confounders (c), using this model:

$$E[Y|a,m,c] = \theta_0 + \theta_1 a + \theta_2 m + \theta_3 am + \theta'_4 c$$

Next, the effect of the intervention on the mediator is determined, using this model:

$$E[M|a,c] = \beta_0 + \beta_1 a + \beta'_2 c$$

A single covariate will be added to both regression models to indicate each study, in order to adjust for possible residual confounding by study differences. Using these models, where θi and βi are the regression coefficients, the natural direct effect (NDE) and natural indirect effect (NIE) are defined as:

$$NDE = \{\theta_1 + \theta_3(\beta_0+\beta_1 a+\beta'_2 c)\}$$

$$NIE = (\theta_2\beta_1 + \theta_3\beta_1 a)$$

For peer review only

and the total effect (TE) is equal to the sum of NDE and NIE[49]. Hence, the percentage mediated will be calculated by dividing NIE by TE and multiply this by 100%.

For knee OA, the mediating effect of upper leg muscle strength, extension deficits and proprioception will initially be analysed separately, using all data available for the IPD data meta-analysis. The effect of each potential mediator will then be evaluated adjusting for the other mediators in a multi-moderator model (Figure 1B). In the latter, a separate linear regression model will be calculated for each mediator[49] and the NIE, hence the percentage mediated, of each mediator can be calculated.

For hip OA, only the effect of muscle strength will be evaluated since this was the only factor indicated as a potential mediator in a systematic review[26]. In all analyses, the mediator will be defined as the absolute change from pre- to post-intervention. Therefore, the outcome measures that will be used will be those measured as close as possible to the end of the intervention period and to the measurement of the mediator(s) under investigation. All analyses will be run with and without stratification for type of exercise intervention.

Sensitivity analysis: Investigation of risk of bias

Effect estimates will be explored using data only from trials deemed to be at low risk of bias from: random sequence generation; allocation concealment; blinded outcome assessment; incomplete outcome data; and trials deemed to be at low risk of bias across all domains.

For peer review only

Unavailable IPD: For trials where IPD were not obtained, we will seek to extract suitable aggregate data from their trial publications and combine these with the IPD trials using suitable statistical methods, to determine whether conclusions remain the same[27]. This is only likely to be possible when examining overall effects, as subgroup differences (interactions) are rarely reported.

Investigation of small study effects: In meta-analyses of 10 trials or more, contour-enhanced funnel plots and tests for asymmetry will be used to investigate small trial effects and the potential for publication bias. Restriction to 10 trials is because there is low power to detect small trial effects with few studies[50].

ETHICS AND DISSEMINATION

Research ethical or governance approval is exempt for this study as no new data are being collected; [51,52]. Findings will be presented at national (UK) and international conferences, and submitted for publication in high-quality peer review journals. We will more broadly disseminate the results to physiotherapists, GPs, practice nurses, orthopaedic surgeons, patients and the general public both nationally and internationally by posting summaries on University websites, placing summaries in local health care settings and sending a summary to relevant groups and organisations for wider dissemination e.g. Osteoarthritis Research Society International (OARSI), the European League Against Rheumatism (EULAR), the American College of Rheumatology (ACR) and Arthritis Research UK. Our PPIE working group will assist in developing plain English summaries and will jointly write articles that will be sent to newspapers, news websites, radio and other news media for wider dissemination.

For peer review only

TABLES AND FIGURES

Table 1: Inclusion/ exclusion criteria

Figure 1: Causal pathway of potential mediators (panel A: single mediator, panel B: multiple mediators)

AUTHORS' CONTRIBUTIONS

MAH, DLB, JR, DvdW, RDR, KD, NEF contributed to the initial conception of the study. MAH, DLB, JR, DvdW, RDR, KD, AL, ALE, HA, KB, JB, KLB, DB, LB, KC, RC, TC, MdR, MD,HPF, SH, RSH, MHR, MVH, CI, JK, IK, CM, SPM, DLP, NS, LAT, RT, CHT, MvM, CW and NEF made a substantial contribution to the design of the work. MAH, DLB and JR drafted the manuscript. MAH, DLB, JR, DvdW, RDR, KD, AL, ALE, HA, KB, JB, KLB, DB, LB, KC, RC, TC, MdR, MD,HPF, SH, RSH, MHR, MVH, CI, JK, IK, CM, SPM, DLP, NS, LAT, RT, CHT, MvM, CW and NEF revised the manuscript and approved the final submission. The OA trial bank steering committee peer reviewed and approved the study protocol. All authors agree to be accountable for all aspects of the work in ensuring that questions related to the accuracy or integrity of any part of the work are appropriately investigated and resolved. The guarantor of the review is NEF.

FUNDING STATEMENT

This work is supported by a Grant from the Chartered Society of Physiotherapy Charitable Trust (Grant number PRF/16/A07), and the National Institute for Health Research (NIHR) School of Primary Care Research (Grant number 531). MAH and DLB are supported by National Institute for Health Research (NIHR) School of Primary Care Research Fellowships. JR received partial funding from a grant of the

For peer review only

Dutch Arthritis Foundation for their center of excellence ‘osteoarthritis in primary care’. KB is funded by an Australian National Health and Medical Research Council Fellowship (#1058440). Musculoskeletal Statistics Unit, The Parker Institute, Bispebjerg and Frederiksberg Hospital (RC) is supported by a core grant from the Oak Foundation (OCAY-13-309). DvdW is a member of PROGRESS Medical Research Council Prognosis Research Strategy (PROGRESS) Partnership (G0902393/99558). KD is part-funded by a Knowledge Mobilisation Research Fellowship (KMRF-2014-03-002) from the NIHR and the NIHR Collaborations for Leadership in Applied Health Research and Care West Midlands. RSH is supported by an Australian Research Council Future Fellowship (FT130100175). NEF, a NIHR Senior Investigator, is supported through an NIHR Research Professorship (NIHR-RP-011-015). The funders did not influence the study design or the writing of this article. The views expressed in this publication are those of the authors and not necessarily those of the NHS, the NIHR or the Department of Health.

COMPETING INTERESTS

None to declare.

DATA SHARING STATEMENT

After this study has been completed, the individual participant data gathered will be deposited with the OA trial bank for the benefit of the wider OA community. Requests for future use of the data will be considered by the OA trial bank, and individual trial principal investigators, as applicable.

REFERENCES

For peer review only

1. National Institute for Health and Care Excellence. Osteoarthritis: care and management in adults. *NICE clinical guideline 177*. Royal College of Physicians, London, 2014.

2. Cross M, Smith E, Hoy D, et al. The global burden of hip and knee osteoarthritis: estimates from the global burden of disease 2010 study. *Ann Rheum Dis* 2014;73:1323-1330.

3. Lawrence RC, Felson DT, Helmick CG, et al. Estimates of the prevalence of arthritis and other rheumatic conditions in the United States. Part II. *Arthritis & Rheum* 2008;58:26-35.

4. Bennell KL, Hall M, Hinman RS. Osteoarthritis year in review 2015: rehabilitation and outcomes. *Osteoarthritis Cartilage* 2016;24:58-70.

5. Brosseau L, Rahman P, Toupin-April K, et al. A systematic critical appraisal for non-pharmacological management of osteoarthritis using the appraisal of guidelines research and evaluation II instrument. *PLoS One* 2014;9(1):e82986.

6. Nelson AE, Allen KD, Golightly YM, et al. A systematic review of recommendations and guidelines for the management of osteoarthritis: The Chronic Osteoarthritis Management Initiative of the U.S. Bone and Joint Initiative. *Semin Arthritis Rheum* 2014;43:701-712.

7. Nicolson PJA, Bennell KL, Dobson FL, et al. Interventions to increase adherence to therapeutic exercise in older adults with low back pain and/or hip/knee osteoarthritis: a systematic review and meta-analysis. *Br J Sports Med* 2017;51:791-799.

8. Fransen M, McConnell S, Harmer AR, et al. Exercise for osteoarthritis of the knee. *Cochrane Database Syst Rev* 2015;1:CD004376. doi:10.1002/14651858.CD004376.

For peer review only

9. Fransen M, McConnell S, Hernandez-Molina G, et al. Exercise for osteoarthritis of the hip. *Cochrane Database Syst Rev* 2014;4:CD007912. doi:10.1002/14651858.CD007912.pub2.

10. Uthman OA, van der Windt DA, Jordan JL, et al. Exercise for lower limb osteoarthritis: systematic review incorporating trial sequential analysis and network meta-analysis. *BMJ* 2013;347:f5555.

11. Christensen R, Henriksen M, Leeds AR, et al. Effect of Weight Maintenance on Symptoms of Knee Osteoarthritis in Obese Patients: A Twelve-Month Randomized Controlled Trial. *Arthritis Care Res* 2015;67:640-650.

12. Foster NE, Thomas E, Barlas P, et al. Acupuncture as an adjunct to exercise based physiotherapy for osteoarthritis of the knee: randomised controlled trial. *BMJ* 2007;335:436.

13. Foster NE, Nicholls E, Holden MA, et al. Improving the effectiveness of exercise therapy for older adults with knee pain: a pragmatic randomised controlled trial (the beep trial). *Physiotherapy* 2015;e404-405.

14. Foster NE, Hill JC, O'Sullivan P, et al. Stratified models of care. *Best Pract Res Clin Rheumatol* 2013;27:649-661.

15. Hill JC, Whitehurst DG, Lewis M, et al. Comparison of stratified primary care management for low back pain with current best practice (STarT Back): a randomised controlled trial. *Lancet* 2011;378:1560-1571.

16. Kraemer HC, Wilson GT, Fairburn CG, et al. Mediators and moderators of treatment effects in randomized clinical trials. *Arch Gen Psychiatry* 2002;59:877-883.

17. Wright AA, Cook CE, Flynn TW, et al. Predictors of response to physical therapy intervention in patients with primary hip osteoarthritis. *Phys Ther* 2011;91:510-524.

For peer review only

18. French HP, Galvin R, Cusack T, et al. Predictors of short-term outcome to exercise and manual therapy for people with hip osteoarthritis. *Phys Ther* 2014;94:31-39.

19. Bennell KL, Dobson F, Roos EM, et al. Influence of biomechanical characteristics on pain and function outcomes from exercise in medial knee osteoarthritis and varus malalignment: exploratory analyses from a randomized controlled trial*. Arthritis Care Res* 2015;67:1281-1288.

20. Kudo M, Watanabe K, Otsubo H, et al. Analysis of effectiveness of therapeutic exercise for knee osteoarthritis and possible factors affecting outcome. *J Orthop Sci* 2013;18:932-939.

21. Knoop J, van der Leeden M, Roorda LD, et al. Knee joint stabilization therapy in patients with osteoarthritis of the knee and knee instability: subgroup analyses in a randomized, controlled trial. *J Rehabil Med* 2014;46:703-707.

22. Knoop J, Dekker J, van der Leeden M, et al. Is the severity of knee osteoarthritis on magnetic resonance imaging associated with outcome of exercise therapy? *Arthritis Care Res* 2014;66:63-68.

23. Foster NE, Thomas E, Hill JC, et al. The relationship between patient and practitioner expectations and preferences and clinical outcomes in a trial of exercise and acupuncture for knee osteoarthritis. *Eur J Pain* 2010;14:402-409.

24. Sun X, Ioannidis JP, Agoritsas T, et al. How to use a subgroup analysis: users' guide to the medical literature. *JAMA* 2014;311:405-11.

25. Juhl C, Christensen R, Roos EM, et al. Impact of exercise type and dose on pain and disability in knee osteoarthritis: a systematic review and meta-regression analysis of randomized controlled trials. *Arthritis Rheumatol* 2014;66(3):622-636.

For peer review only

26. Runhaar J, Luijsterburg P, Dekker J, et al. Identifying potential working mechanisms behind the positive effects of exercise therapy on pain and function in osteoarthritis; a systematic review. *Osteoarthritis Cartilage* 2015;23:1071-1082.

27. Riley RD, Lambert PC, Abo-Zaid G. Meta-analysis of individual participant data: rationale, conduct, and reporting. *BMJ* 2010;340:c221.

28. Tierney JF, Vale C, Riley R, et al. Individual Participant Data (IPD) Meta-analyses of Randomised Controlled Trials: Guidance on Their Use. *PLoS Med* 2015;12:e1001855.

29. Debray TP, Moons KG, van Valkenhoef G, et al. Get real in individual participant data (IPD) meta-analysis: a review of the methodology. *Res Synth Methods* 2015;6:293-309.

30. Stewart LA, Clarke M, Rovers M, et al. Preferred Reporting Items for a Systematic Review and Meta-analysis of Individual Participant Data: The PRISMA-IPD Statement. *JAMA* 2015;313:1657-1665.

31. INVOLVE. Public involvement in research: values and principles framework, INVOLVE: Eastleigh, 2015.

32. van Middelkoop M, Dziedzic KS, Doherty M, et al. Individual patient data meta-analysis of trials investigating the effectiveness of intra-articular glucocorticoid injections in patients with knee or hip osteoarthritis: an OA Trial Bank protocol for a systematic review. *Syst Rev* 2013;2:54.

33. van Middelkoop M, Arden NK, Atchia I, et al. The OA Trial Bank: meta-analysis of individual patient data from knee and hip osteoarthritis trials show that patients with severe pain exhibit greater benefit from intra-articular glucocorticoids. *Osteoarthritis Cartilage* 2016;S1063-4584(16)01002-5.

For peer review only

34. Brosseau L, Taki J, Desjardins B, et al. The Ottawa panel clinical practice guidelines for the management of knee osteoarthritis. Part one: introduction, and mind-body exercise programs. *Clin Rehabil* 2017;31:582-595.

35. Kontopantelis E, Springate DA, Parisi R et al. Simulation-Based Power Calculations for Mixed Effects Modeling: ipdpower in Stata. *Journal of Statistical Software* 2016;74:25.

36. Hróbjartsson A. Why did it take 19 months to retrieve clinical trial data from a non-profit organisation? *BMJ* 2013;347.

37. Ahmed I, Sutton AJ and Riley RD. Assessment of publication bias, selection bias and unavailable data in meta-analyses using individual participant data: a database survey. *BMJ* 2012;344:d7762.

38. Luyten FP, Denti M, Filardo G, et al. Definition and classification of early osteoarthritis of the knee. *Knee Surg Sports Traumatol Arthrosc* 2012;20:401-406.

39. Cochrane Musculoskeletal Group. URL: http://musculoskeletal.cochrane.org/proposed-outcomes (accessed 5th May 2017).

40. Higgins JPT, Green S (editors). *Cochrane Handbook for Systematic Reviews of Interventions* Version 5.1.0 [updated March 2011]. *The Cochrane Collaboration* 2011 www.handbook.cochrane.org (accessed 5th May 2017).

41. Hartung J, Knapp G. A refined method for the meta-analysis of controlled clinical trials with binary outcome. *Stat Med* 2001;20:3875-89.

42. Hartung J, Knapp G. On tests of the overall treatment effect in meta-analysis with normally distributed responses. *Stat Med* 2001;20:1771-82.

43. Quartagno M, Carpenter J.R. Multiple imputation for IPD meta-analysis: allowing for heterogeneity and studies with missing covariates. *Stats in Med* 2016;35:2938-54.

For peer review only

704 44. Carpenter JR, Kenward M. Multiple Imputation and its Application. Chichester: John Wiley & Sons 2013.

706 45. SAS Institute Inc. SAS/STAT_ 9.3 user's guide. Cary, NC: SAS Institute Inc, 2011.

708 46. StataCorp, Stata Statistical Software: Release 14. 2015, StataCorp LP: College Station, TX.

710 47. Jones AP, Riley RD, Williamson PR, et al. Meta-analysis of individual patient data versus aggregate data from longitudinal clinical trials. *Clin Trials* 2009;6:16-27.

712 48. Riley RD, Price MJ, Jackson D, et al. Multivariate meta-analysis using individual participant data. *Res Synth Methods* 2015;6:157-174.

714 49. VanderWeele TJ, Vansteelandt S. Mediation Analysis with Multiple Mediators. *Epidemiol Method* 2014;2:95-115.

716 50. Sterne JA, Sutton AJ, Ioannidis JP, et al. Recommendations for examining and interpreting funnel plot asymmetry in meta-analyses of randomised controlled trials. *BMJ* 2011;343:d4002.

719 51. Taichman DB, Backus J, Baethge C, et al. Sharing Clinical Trial Data: A Proposal From the International Committee of Medical Journal Editors. *JAMA* 2016;315:467-468.

722 52. Menikoff J. Letter from Jerry Menikoff, MD, JD. Director, Office for Human Research Protections, to ICMJE Secretairat. March 7, 2017. http://icmje.org/news-and-editorials/menikoff_icmje_questions_20170307.pdf (accessed 10th July 2017).

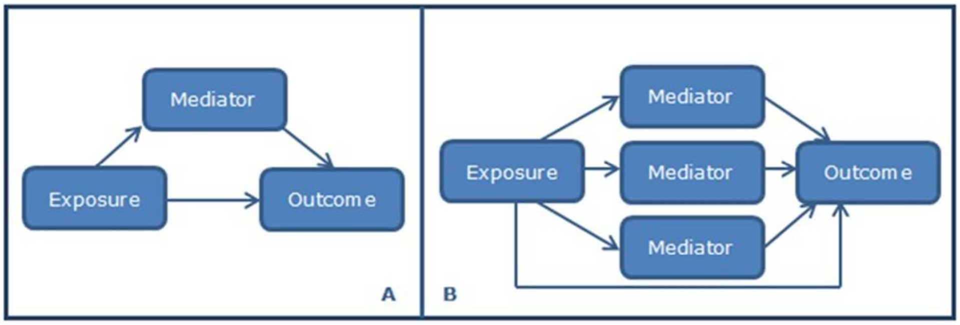

Figure 1: Causal pathway of potential mediators (panel A: single mediator, panel B: multiple mediators)

165x60mm (120 x 120 DPI)

For peer review only

Appendix 1: Full search strategies for Medline and Embase

Medline search	Embase search
1. randomized controlled trial.pt.<br>2. controlled clinical trial.pt.<br>3. randomized.ab.<br>4. placebo.ab.<br>5. drug therapy.fs.<br>6. randomly.ab.<br>7. trial.ab.<br>8. groups.ab.<br>9. therapy.fs.<br>10. 1 or 2 or 3 or 4 or 5 or 6 or 7 or 8 or 9<br>11. limit 10 to humans<br>12. exp Exercise Movement Techniques/ or exp Exercise/ or exp Exercise Therapy/<br>13. ((strength$ or isometric$ or isotonic$ or isokinetic$ or endurance or weight$) adj5 train$).ti,ab.<br>14. (resistance adj5 (exercis$ or train$)).ti,ab.<br>15. ((physical$ or motion$) adj5 (fit$ or therap$)).ti,ab.<br>16. (physical$ adj5 activ$).ti,ab.<br>17. physiotherap$.ti,ab.<br>18. kinesiotherap$.ti,ab.<br>19. rehab$.mp.<br>20. exp Physical Fitness/<br>21. (walk$ or jog$ or run$ or cycl$ or swim$ or treadmill$ or gym$ or bicycl$ or skat$ or row$).ti,ab.<br>22. exp Sports/<br>23. sport$.ti,ab.<br>24. exercise$.ti,ab.<br>25. aerobic$.ti,ab.<br>26. yoga.mp. or (tai adj1 (chi or ji)).ti,ab. [mp=title, original title, abstract, name of substance word, subject heading word, unique identifier]<br>27. danc$.ti,ab.<br>28. (aqua$ or water).ti,ab.<br>29. hydro$.ti,ab.<br>30. exp Hydrotherapy/<br>31. (stretch$ or flexib$ or balanc$ or propriocept$).ti,ab.<br>32. (circuit$ adj1 train$).ti,ab.<br>36. 12 or 13 or 14 or 15 or 16 or 17 or 18 or 19 or 20 or 21 or 22 or 23 or 24 or 25 or 26 or 27 or 28 or 29 or 30 or 31 or 32<br>37. exp Osteoarthritis/<br>38. osteoarthr$.ti,ab.<br>39. OA.ti,ab.<br>40. degenerative arthritis.mp.<br>41. arthrosis.mp.<br>42. 37 or 38 or 40 or 41<br>43. exp Knee Joint/ or exp Knee/<br>44. exp Hip Joint/ or exp Hip/<br>45. (knee$ or hip$).ti,ab.<br>46. 43 or 44 or 45<br>47. 42 and 46<br>48. ((knee$ or hip$) adj5 pain).ti,ab.<br>49. 47 or 48<br>50. 11 and 36 and 49	1. crossover procedure/<br>2. double-blind procedure/<br>3. randomized controlled trial/<br>4. single-blind procedure/<br>5. random$.ti,ab.<br>6. factorial$.ti,ab.<br>7. crossover$.ti,ab.<br>8. cross over$.ti,ab.<br>9. placebo$.ti,ab.<br>10. (doubl$ adj blind$).ti,ab.<br>11. (singl$ adj blind$).ti,ab.<br>12. assign$.ti,ab.<br>13. allocat$.ti,ab.<br>14. volunteer$.ti,ab.<br>15. 1 or 2 or 3 or 4 or 5 or 6 or 7 or 8 or 9 or 10 or 11 or 12 or 13 or 14<br>16. exp exercise/<br>17. exp kinesiotherapy/<br>18. fitness/<br>19. ((strength$ or isometric$ or isotonic$ or isokinetic$ or endurance or weight$) adj5 train$).ti,ab.<br>20. (resistance adj5 (exercis$ or train$)).ti,ab.<br>21. ((physical$ or motion$) adj5 (fit$ or therap$)).ti,ab.<br>22. (physical$ adj5 activ$).ti,ab.<br>23. physiotherap$.ti,ab.<br>24. kinesiotherap$.ti,ab.<br>25. rehab$.mp.<br>26. (walk$ or jog$ or run$ or cycl$ or swim$ or treadmill$ or gym$ or bicycl$ or skat$ or row$).ti,ab.<br>27. sport$.ti,ab.<br>28. exp SPORT/<br>29. exercise$.ti,ab.<br>30. aerobic$.ti,ab.<br>31. (yoga or (tai adj1 (chi or ji))).ti,ab.<br>32. danc$.ti,ab.<br>33. (aqua$ or water).ti,ab.<br>34. hydro$.ti,ab.<br>35. exp hydrotherapy/<br>36. (stretch$ or flexib$ or balanc$ or propriocept$).ti,ab.<br>37. (circuit$ adj1 train$).ti,ab.<br>38. or/16–37<br>39. exp OSTEOARTHRITIS/<br>40. osteoarthr$.ti,ab.<br>41. OA.ti,ab.<br>42. degenerative arthritis.mp.<br>43. arthrosis.mp.<br>44. 39 or 40 or 41 or 42 or 43<br>45. exp knee/<br>46. exp HIP/<br>47. (knee$ or hip$).ti,ab.<br>48. 45 or 46 or 47<br>49. 44 and 48<br>50. ((knee$ or hip$) adj5 pain).ti,ab.<br>51. 49 or 50<br>52. 15 and 38 and 51<br>53. limit 52 to human

For peer review only

For peer review only

PRISMA-P 2015 Checklist – STEER OA

This checklist has been adapted for use with protocol submissions to *Systematic Reviews* from Table 3 in Moher D et al: Preferred reporting items for systematic review and meta-analysis protocols (PRISMA-P) 2015 statement. *Systematic Reviews* 2015 **4**:1

For peer review only

Section/topic	#	Checklist item	Information reported Yes	Information reported No	Line number(s)
**ADMINISTRATIVE INFORMATION**					
**Title**					
Identification	1a	Identify the report as a protocol of a systematic review	☒	☐	2
Update	1b	If the protocol is for an update of a previous systematic review, identify as such	☒	☐	2
**Registration**	2	If registered, provide the name of the registry (e.g., PROSPERO) and registration number in the Abstract	☒	☐	130
**Authors**					
Contact	3a	Provide name, institutional affiliation, and e-mail address of all protocol authors; provide physical mailing address of corresponding author	☒	☐	5-99
Contributions	3b	Describe contributions of protocol authors and identify the guarantor of the review	☒	☐	539-545
**Amendments**	4	If the protocol represents an amendment of a previously completed or published protocol, identify as such and list changes; otherwise, state plan for documenting important protocol amendments	☐	☒	
**Support**					
Sources	5a	Indicate sources of financial or other support for the review	☒	☐	548-566
Sponsor	5b	Provide name for the review funder and/or sponsor	☒	☐	548-550
Role of sponsor/funder	5c	Describe roles of funder(s), sponsor(s), and/or institution(s), if any, in developing the protocol	☒	☐	564-566
**INTRODUCTION**					
**Rationale**	6	Describe the rationale for the review in the context of what is already known	☒	☐	147-226
**Objectives**	7	Provide an explicit statement of the question(s) the review will address with reference to	☒	☐	236-255

BioMed Central
The Open Access Publisher

2

Section/topic	#	Checklist item	Information reported		Line number(s)
			Yes	No	
		participants, interventions, comparators, and outcomes (PICO)			
**METHODS**					
**Eligibility criteria**	8	Specify the study characteristics (e.g., PICO, study design, setting, time frame) and report characteristics (e.g., years considered, language, publication status) to be used as criteria for eligibility for the review	☒	☐	286-297, 299, table 1
**Information sources**	9	Describe all intended information sources (e.g., electronic databases, contact with study authors, trial registers, or other grey literature sources) with planned dates of coverage	☒	☐	272-282
**Search strategy**	10	Present draft of search strategy to be used for at least one electronic database, including planned limits, such that it could be repeated	☒	☐	282, appendix 1
STUDY RECORDS					
Data management	11a	Describe the mechanism(s) that will be used to manage records and data throughout the review	☒	☐	329-344
Selection process	11b	State the process that will be used for selecting studies (e.g., two independent reviewers) through each phase of the review (i.e., screening, eligibility, and inclusion in meta-analysis)	☒	☐	295-297, 306-310
Data collection process	11c	Describe planned method of extracting data from reports (e.g., piloting forms, done independently, in duplicate), any processes for obtaining and confirming data from investigators	☒	☐	330-344
**Data items**	12	List and define all variables for which data will be sought (e.g., PICO items, funding sources), any pre-planned data assumptions and simplifications	☒	☐	347-362
**Outcomes and prioritization**	13	List and define all outcomes for which data will be sought, including prioritization of main and additional outcomes, with rationale	☒	☐	302-326, 364-379
**Risk of bias in individual studies**	14	Describe anticipated methods for assessing risk of bias of individual studies, including whether this will be done at the outcome or study level, or both; state how this information will be used in data synthesis	☒	☐	393-402
DATA					
**Synthesis**	15a	Describe criteria under which study data will be quantitatively synthesized	☒	☐	382-390
	15b	If data are appropriate for quantitative synthesis, describe planned summary measures, methods of handling data, and methods of combining data from studies, including any planned exploration of consistency (e.g., I^2, Kendall's tau)	☒	☐	405-422
	15c	Describe any proposed additional analyses (e.g., sensitivity or subgroup analyses, meta-	☒	☐	504-516

BioMed Central The Open Access Publisher

For peer review only

Section/topic	#	Checklist item	Information reported Yes	No	Line number(s)
		regression)			
	15d	If quantitative synthesis is not appropriate, describe the type of summary planned	☐	☒	
**Meta-bias(es)**	16	Specify any planned assessment of meta-bias(es) (e.g., publication bias across studies, selective reporting within studies)	☒	☐	393-402
**Confidence in cumulative evidence**	17	Describe how the strength of the body of evidence will be assessed (e.g., GRADE)	☐	☒	

For peer review only

BioMed Central
The Open Access Publisher

BMJ Open

Subgrouping and TargetEd Exercise pRogrammes for knee and hip OsteoArthritis (STEER OA): A systematic review update and individual participant data meta-analysis protocol

Journal:	*BMJ Open*
Manuscript ID	bmjopen-2017-018971.R1
Article Type:	Protocol
Date Submitted by the Author:	09-Oct-2017

Complete List of Authors:	Holden, Melanie; Keele University, Arthritis Research UK Primary Care Centre, Research Institute of Primary Care and Health Sciences<br>Burke, Danielle; Keele University, Arthritis Research UK Primary Care Centre, Research Institute of Primary Care and Health Sciences<br>Runhaar, Jos; Erasmus University Medical Center Rotterdam, Department of General Practice<br>van der Windt, Danielle; Keele University, Arthritis Research UK Primary Care Centre, Research Institute of Primary Care and Health Sciences<br>Riley, Richard; Keele University, Arthritis Research UK Primary Care Centre, Research Institute of Primary Care and Health Sciences<br>Dziedzic, Krysia; Keele University, Arthritis Research UK Primary Care Centre, Research Institute of Primary Care and Health Sciences<br>Legha, Amardeep; Keele University, Arthritis Research UK Primary Care Centre, Research Institute of Primary Care and Health Sciences<br>Evans, Amy; Keele University, Arthritis Research UK Primary Care Centre, Research Institute of Primary Care and Health Sciences<br>Abbott, J. Haxby; University of Otago, Centre for Musculoskeletal Outcomes Research, Orthopaedic Surgery Section, Department of Surgical Sciences, Dunedin School of Medicine<br>Baker, Kristin; Boston University, Sargent College<br>Brown, Jenny; Keele University, Research User Group, Arthritis Research UK Primary Care Centre, Research Institute of Primary Care and Health Sciences<br>Bennell, Kim; University of Melbourne, Centre for Health, Exercise & Sports Medicine, Department of Physiotherapy<br>Bossen, Daniël; Amsterdam University of Applied Sciences, ACHIEVE Centre of Expertise, Faculty of Health; University of Amsterdam, Coronel Institute of Occupational Health, Academic Medical Center<br>Brosseau, Lucie; University of Ottawa, Faculty of Health Sciences, School of Rehabilitation Sciences<br>Chaipinyo, Kanda; Srinakharinwirot University, Division of Physical Therapy, Faculty of Health Science<br>Christensen, Robin; Frederiksberg and Bispebjerg Hospital, Musculoskeletal Statistics Unit, The Parker Institute, Department of Rheumatology<br>Cochrane, Tom; University of Canberra, Centre for Research Action in Public Health<br>de Rooij, Mariette; Reade, Amsterdam Rehabilitation Research Centre, Centre for Rehabilitation and Rheumatology<br>Doherty, Michael ; University of Nottingham, Academic Rheumatology

French, Helen; Royal College of Surgeons in Ireland, School of Physiotherapy
Hickson, Sheila; Keele University, Research User Group, Arthritis Research UK Primary Care Centre, Research Institute of Primary Care and Health Sciences
Hinman, Rana S.; University of Melbourne, Centre for Health, Exercise & Sports Medicine, Department of Physiotherapy
Hopman-Rock, M; TNO Netherlands Organisation for Applied Scientific Research; VU University Medical Center, The Netherlands Department of Public and Occupational Health, EMGO Institute for Health and Care Research
Hurley, Mike; St George's University of London and Kingston University, Faculty of Health, Social Care and Education; Health Innovation Network South London
Ingram, Carol; Keele University, Research User Group, Arthritis Research UK Primary Care Centre, Research Institute of Primary Care and Health Sciences
Knoop, J; Reade, Amsterdam Rehabilitation Research Centre, Centre for Rehabilitation and Rheumatology
Krauss, Inga; University Hospital of Tübingen, Department of Sports Medicine, Medical Clinic
McCarthy, Chris; Manchester Metropolitan University, Manchester Movement Unit, Manchester School of Physiotherapy
Messier, Stephen; Wake Forest University, J.B. Snow Biomechanics Laboratory, Department of Health and Exercise Science, Worrell Professional Center
Patrick, Donald; University of Washington, Department of Health Services
Sahin, Nilay; Balikesir Universitesi, Department of Physical Medicine and Rehabilitation, Medical Faculty
Talbot, Laura; University of Tennessee Health Science Center, College of Medicine, Department of Neurology
Taylor, Robert; Keele University, Research User Group, Arthritis Research UK Primary Care Centre, Research Institute of Primary Care and Health Sciences
Teirlinck, Carolien; Erasmus University Medical Center Rotterdam, Department of General Practice
van Middelkoop, Marienke; Erasmus University Medical Center Rotterdam, Department of General Practice
Walker, Christine; Keele University, Research User Group, Arthritis Research UK Primary Care Centre, Research Institute of Primary Care and Health Sciences
Foster, Nadine; Keele University, Arthritis Research UK Primary Care Centre, Research Institute of Primary Care and Health Sciences

**Primary Subject Heading**:	Rheumatology
Secondary Subject Heading:	Rehabilitation medicine
Keywords:	Musculoskeletal disorders < ORTHOPAEDIC & TRAUMA SURGERY, Hip < ORTHOPAEDIC & TRAUMA SURGERY, Knee < ORTHOPAEDIC & TRAUMA SURGERY, osteoarthritis, exercise

SCHOLARONE™
Manuscripts

TITLE: Subgrouping and TargetEd Exercise pRogrammes for knee and hip OsteoArthritis (STEER OA): A systematic review update and individual participant data meta-analysis protocol

AUTHORS:

1. Melanie A Holden[1] m.holden@keele.ac.uk
2. Danielle L Burke[1] d.burke@keele.ac.uk
3. Jos Runhaar[2] j.runhaar@erasmusmc.nl
4. Danielle van Der Windt[1] d.van.der.windt@keele.ac.uk
5. Richard D Riley[1] r.riley@keele.ac.uk
6. Krysia Dziedzic[1] k.s.dziedzic@keele.ac.uk
7. Amardeep Legha[1] a.legha@keele.ac.uk
8. Amy L Evans[1] a.evans1@keele.ac.uk
9. Haxby Abbott[3] haxby.abbott@otago.ac.nz
10. Kristin Baker[4] krbaker@bu.edu
11. Jenny Brown[5]
12. Kim L Bennell[6] k.bennell@unimelb.edu.au
13. Daniël Bossen[7,8] d.bossen@hva.nl
14. Lucie Brosseau[9] lbrossea@uottawa.ca
15. Kanda Chaipinyo[10] kanda@g.swu.ac.th
16. Robin Christensen[11] robin.christensen@regionh.dk
17. Tom Cochrane[12] Tom.Cochrane@canberra.edu.au
18. Mariette de Rooij[13] m.d.rooij@reade.nl
19. Michael Doherty[14] Michael.Doherty@nottingham.ac.uk
20. Helen P French[15] hfrench@rcsi.ie

For peer review only

For peer review only - http://bmjopen.bmj.com/site/about/guidelines.xhtml

21. Sheila Hickson[5]

22. Rana S Hinman[6] ranash@unimelb.edu.au

23. Marijke Hopman-Rock[16,17] m.hopman@vumc.nl

24. Michael V Hurley[18,19] Michael.Hurley@sgul.kingston.ac.uk

25. Carol Ingram[5]

26. Jesper Knoop[13] j.knoop@reade.nl

27. Inga Krauß[20] Inga.Krauss@med.uni-tuebingen.de

28. Chris McCarthy[21] cmccarthy@mmu.ac.uk

29. Stephen P Messier[22] messier@wfu.edu

30. Donald L. Patrick[23] donald@uw.edu

31. Nilay Sahin[24] nilaysahin@gmail.com

32. Laura A Talbot[25] ltalbot@uthsc.edu

33. Robert Taylor[5]

34. Carolien H Teirlinck[2] c.teirlinck@erasmusmc.nl

35. Marienke van Middelkoop[2] m.vanmiddelkoop@erasmusmc.nl

36. Christine Walker[5]

37. Nadine E Foster[1] n.foster@keele.ac.uk

In collaboration with the OA Trial Bank

Affiliations:

1. Arthritis Research UK Primary Care Centre, Research Institute of Primary Care and Health Sciences, Keele University, Keele, United Kingdom.

2. Erasmus University Medical Center Rotterdam, Department of General Practice, Rotterdam, the Netherlands.

For peer review only

3. Centre for Musculoskeletal Outcomes Research, Orthopaedic Surgery Section, Department of Surgical Sciences, Dunedin School of Medicine, University of Otago, Dunedin, New Zealand.

4. Sargent College, Boston University, Boston, United States of America.

5. Research User Group, Arthritis Research UK Primary Care Centre, Research Institute of Primary Care and Health Sciences, Keele University, Keele, United Kingdom.

6. Centre for Health, Exercise & Sports Medicine, Department of Physiotherapy, University of Melbourne, Melbourne, Australia.

7. ACHIEVE Centre of Expertise, Faculty of Health, Amsterdam University of Applied Sciences, Amsterdam, the Netherlands.

8. Coronel Institute of Occupational Health, Academic Medical Center, University of Amsterdam, Amsterdam, the Netherlands.

9. Faculty of Health Sciences, School of Rehabilitation Sciences, University of Ottawa, Ottawa, Canada.

10. Division of Physical Therapy, Faculty of Health Science, Srinakharinwirot University, Bangkok, Thailand.

11. Musculoskeletal Statistics Unit, The Parker Institute, Bispebjerg and Frederiksberg Hospital, Copenhagen, Denmark.

12. Centre for Research & Action in Public Health, University of Canberra, Canberra, Australia.

13. Amsterdam Rehabilitation Research Center, Reade, Centre for Rehabilitation and Rheumatology, Amsterdam, the Netherlands.

14. Academic Rheumatology, University of Nottingham, Nottingham, United Kingdom

For peer review only

For peer review only - http://bmjopen.bmj.com/site/about/guidelines.xhtml

15. School of Physiotherapy, Royal College of Surgeons in Ireland, Dublin, Ireland.

16. TNO Netherlands Organisation for Applied Scientific Research, Leiden, the Netherlands.

17. The Netherlands Department of Public and Occupational Health, EMGO Institute for Health and Care Research, VU University Medical Center, Amsterdam, the Netherlands.

18. Faculty of Health, Social Care and Education, St George's University of London and Kingston University, London, United Kingdom.

19. Health Innovation Network South London, London, United Kingdom.

20. Department of Sports Medicine, Medical Clinic, University Hospital of Tübingen, Tübingen, Germany.

21. Manchester Movement Unit, Manchester School of Physiotherapy, Manchester Metropolitan University, United Kingdom.

22. J.B. Snow Biomechanics Laboratory, Department of Health and Exercise Science, Worrell Professional Center, Wake Forest University, Winston Salem, United States of America.

23. Department of Health Services, University of Washington, Washington, United States of America.

24. Department of Physical Medicine and Rehabilitation, Medical Faculty, Balıkesir University, Balıkesir, Turkey

25. University of Tennessee Health Science Center, College of Medicine, Department of Neurology, Memphis, United States of America.

For peer review only

Corresponding author: Dr Melanie Holden, Arthritis Research UK Primary Care Centre, Research Institute of Primary Care and Health Sciences, Keele University, Keele, Staffordshire, ST5 5BG. Email: m.holden@keele.ac.uk, Telephone: (+44) 01782 734720

WORD COUNT: 3951

ABSTRACT

Introduction: Knee and hip osteoarthritis (OA) is a leading cause of disability worldwide. Therapeutic exercise is a recommended core treatment for people with knee and hip OA, however, the observed effect sizes for reducing pain and improving physical function are small to moderate. This may be due to insufficient targeting of exercise to subgroups of people who are most likely to respond, and/or sub-optimal content of exercise programmes. This study aims to identify a) subgroups of people with knee and hip OA that do/ do not respond to therapeutic exercise, and to different types of exercise, and b) mediators of the effect of therapeutic exercise for reducing pain and improving physical function. This will enable optimal targeting and refining the content of future exercise interventions.

Methods and analysis: Systematic review and individual participant data meta-analyses. A previous comprehensive systematic review will be updated to identify randomised controlled trials that compare the effects of therapeutic exercise for people with knee and hip OA on pain and physical function, to a non-exercise control. Lead authors of eligible trials will be invited to share individual participant data. Trial- and participant-level characteristics (for baseline variables and

For peer review only

outcomes) of included studies will be summarised. Meta-analyses will use a two-stage approach, where effect estimates are obtained for each trial and then synthesised using a random effects model (to account for heterogeneity). All analyses will be on an intention-to-treat principle and all summary meta-analysis estimates will be reported as standardised mean differences with 95% confidence intervals. **Ethics and dissemination:** Research ethical or governance approval is exempt as no new data are being collected and no identifiable participant information will be shared. Findings will be disseminated via national and international conferences, publication in peer-reviewed journals, and summaries posted on websites accessed by the public and clinicians. **REGISTRATION DETAILS:** PROSPERO: CRD42017054049

STRENGTHS AND LIMITATIONS OF THIS STUDY

1. This will be the first study in the field of therapeutic exercise and osteoarthritis to combine individual participant data from existing randomised controlled trials.
2. Combining individual participant data from existing trials will increase the power to identify who benefits most from therapeutic exercise, and to identify underlying mechanisms of action.
3. Individual participant data meta-analyses facilitates standardised analyses across studies, allows direct derivation of desired information independent of significance or reporting, enables subgroup effects and interactions (differences in effects between subgroups) to be examined, and may provide more outcomes than were considered in a single original publication.

For peer review only

4. A disadvantage to completing individual participant data meta-analyses is the time required to complete them, including obtaining, checking, and combining the data.

For peer review only

For peer review only - http://bmjopen.bmj.com/site/about/guidelines.xhtml

INTRODUCTION

Osteoarthritis (OA) can be defined as a clinical syndrome of joint pain accompanied by varying degrees of functional limitation and reduced quality of life[1]. OA, particularly of the knee and hip, is one of the leading causes of disability worldwide, with an estimated global age-standardised prevalence of 3.8% (95% confidence interval (CI) 3.6% to 4.1%) for knee OA and 0.85% (95% CI 0.74% to 1.02%) for hip OA[2]. The burden of OA will increase as the population ages and the prevalence of obesity rises[2,3].

No cure currently exists for OA and as such treatment strategies aim to reduce pain and improve physical function, and enhance quality of life[4]. Clinical guidelines, including the National Institute for Health and Care Excellence (NICE) OA guidelines[1], consistently recommend therapeutic exercise as a core treatment for people with knee and hip OA[5,6]. Therapeutic exercise involves participation in physical activity that is planned, structured, repetitive and purposeful for the improvement or maintenance of a specific health condition such as OA[7]. It encompasses general aerobic exercise, strengthening, flexibility, balance or body-region specific exercises[7]. Although both general (aerobic) exercise and strengthening exercise are recommended for people with knee and hip OA, limited information is available on the optimal prescription of therapeutic exercise (e.g. the optimal type, dose, intensity, and setting of exercise, and how best to progress exercise)[1]. Numerous systematic reviews and meta-analyses support the role of therapeutic exercise for knee and hip OA, consistently demonstrating that it can reduce pain and improve physical function[8-10]. However, results from randomised

For peer review only

For peer review only - http://bmjopen.bmj.com/site/about/guidelines.xhtml

controlled trials (RCTs) show the observed effect sizes from exercise interventions are small to moderate, can decline over time, and only approximately 50% of participants achieve a clinically important treatment response[11-13]. The modest average benefits obtained from therapeutic exercise could be due to the inclusion of subgroups of people who are unlikely to benefit from exercise, and thus the overall effect is closer to the null than if the trial had been solely undertaken in those that are likely to benefit[4]. Better targeting of exercise treatments for people with knee and hip OA could potentially lead to improved treatment effects and patient outcomes, as well as more efficient use of healthcare services, in a similar way as demonstrated for low back pain[14,15]. Such an approach requires the identification of subgroups who are likely to respond better to therapeutic exercise than others.

Little previous research has examined whether outcomes from exercise for OA vary for subgroups of people defined by individual-level characteristics (treatment effect moderators)[16]. Exploratory secondary analyses of some RCTs suggest a range of potential moderators of the effects of exercise, including age[17], sex[18], obesity[19], pain severity and duration[17,18], functional ability[18], strength[20,21], knee malalignment[19,20], severity of joint damage[22], anxiety and depression[18], and treatment outcome expectations[23]. However, post hoc analyses have low statistical power to detect significant subgroup effects, and are at high risk of yielding spurious findings due to multiple testing[24].Although these exploratory subgroup analyses are inconclusive, they add credence to the hypothesis that not all people with knee and hip OA respond similarly to exercise, with variability in effects related to individual-level characteristics.

For peer review only - http://bmjopen.bmj.com/site/about/guidelines.xhtml

Modest average benefits of therapeutic exercise in people with knee and hip OA may additionally be explained by sub-optimal content of exercise programmes. Systematic reviews have identified various characteristics of exercise programmes that appear to be associated with larger effects, but with conflicting results[10,25]. Treatment mediators (causal links between treatment and outcome[16]) of therapeutic exercise on OA symptoms are largely unknown, making it difficult to design therapeutic exercise programmes for optimal effects on symptoms. If true mediators were identified and targeted, the positive effects of therapeutic exercise may be improved. Increased muscle strength, decreased extension deficits and improved proprioception have been identified as potential working mechanisms for the positive effect of therapeutic exercise for knee OA, and increased muscle strength for hip OA[26]. However, meta-analyses at the study-level (using aggregated study results)[26] have been prone to study-level confounding regarding the identification of individual-level effects[25,26].

The investigation of individual response to exercise treatment, and the identification of factors that may cause differential response to such treatment or to particular components of exercise therapy, requires individual-level data analysis. To our knowledge, this type of trial data pooling and analysis has not yet been completed in the field of therapeutic exercise and OA. Although several systematic reviews are available[8-10], none use individual participant data (IPD). Given there are now over 60 RCTs of exercise for knee and hip OA[10] it is timely to combine IPD from these existing trials. This will increase the power to identify who benefits most from

For peer review only - http://bmjopen.bmj.com/site/about/guidelines.xhtml

therapeutic exercise, and to identify underlying mechanisms of action [27]. IPD meta-analysis facilitates standardised analyses across studies, allows direct derivation of desired information independent of significance or reporting, enables subgroup effects and interactions (differences in effects between subgroups) to be examined, and may provide longer follow-up, more participants, and more outcomes than were considered in the original publication[27]. Therefore IPD meta-analyses are potentially more reliable than traditional aggregate data meta-analyses for the identification of treatment effect moderators, may lead to different conclusions, and may produce more clinically relevant results[27].

AIM

To identify a) subgroups of people with knee and hip OA that respond/ do not respond to therapeutic exercise and to different types of exercise (effect moderators), and b) mediators of the effect of therapeutic exercise for reducing pain and improving physical function, to facilitate better targeting of future exercise interventions and refine exercise program content.

Specific analytic objectives

1. Determine the short- (12 weeks), medium- (6 months) and long-term (1 year) overall effects of therapeutic exercise on pain and physical function for people with knee and hip OA compared to a non-exercise control.

2. Determine which study-level characteristics of therapeutic exercise interventions are associated with improved overall effects on pain and physical function in people

For peer review only

with knee and hip OA, including the type, intensity, duration, setting and deliverer of exercise.

3. Identify individual-level characteristics of people with knee and hip OA that are associated with the short-, medium-, and long-term effects of therapeutic exercise on pain and physical function.

4. Identify individual-level characteristics of people with knee and hip OA that are associated with the effects of different characteristics of therapeutic exercise interventions; including the type, intensity, duration, setting, and deliverer of exercise.

5. Investigate the effect estimates for objectives 1 to 4 in subgroups of people with only knee OA and only hip OA to examine whether they differ by joint site.

6. Evaluate the mediating effects of muscle strength (for people with knee and hip OA), proprioception (for people with knee OA) and extension deficits (for people with knee OA) in the association between therapeutic exercise and pain and physical function. The effects of individual and combined mediators will be explored.

METHODS AND ANALYSIS

We will update a previous systematic review[10] to identify relevant RCTs and after agreement from trial leads, undertake an IPD meta-analysis. Our systematic review and meta-analysis will be completed in accordance with methods advocated by the Cochrane IPD meta-analysis group[28,29], and reported according to PRISMA-IPD guidance[30]. Five members of the Research User Group at the Research Institute of Primary Care and Health Sciences, Keele University, have formed a Patient and Public Involvement and Engagement (PPIE) working group for this study. In line with

For peer review only

For peer review only - http://bmjopen.bmj.com/site/about/guidelines.xhtml

INVOLVE[31] there will be PPIE involvement at every stage of the project. We will work in collaboration with the OA Trial Bank (www.oatrialbank.com), an initiative established in 2010 to collect and analyse IPD of published RCTs in OA[32,33]. The final IPD database will be deposited with the OA Trial Bank for the benefit of the wider OA research community.

PHASE 1: TRIAL IDENTIFICATION

We previously conducted a systematic review that identified 60 RCTs of exercise interventions for people with knee and hip OA that are relevant for inclusion within this study[10]. We will update this review. The search strategy previously developed will be re-run from the date of the previous search (March 2012) in the following electronic databases: Medline, Embase, CINAHL, AMED, HMIC (Health Management Information Consortium), Cochrane Database of Systematic Reviews, Cochrane Controlled Clinical Trials (CENTRAL), DARE (Database of Reviews of Effectiveness), NHS EED (Economic Evaluations Database), and Web of Science. Bibliographies of relevant review articles and included articles will be examined for additional potentially relevant trials. There will be no language restriction. Full search strategies for Medline and Embase are shown in Appendix 1.

Study Selection

Full details of the study selection criteria are listed in Table 1. In summary, we will evaluate RCTs against the following inclusion criteria:

Study population: Adults age 45 years and above with knee or hip OA;

Intervention: Any land or water based therapeutic exercise intervention regardless

For peer review only - http://bmjopen.bmj.com/site/about/guidelines.xhtml

of content, duration, frequency, or intensity; ***Comparator:*** Other forms of exercise or no exercise control group; ***Outcome measure:*** At least one measure of self-reported pain or physical function; ***Study design:*** RCTs. Trials will be excluded if they concern pre- or post-operative therapeutic exercise, when exercise is combined with interventions other than advice/ education/ self-management/ motivational techniques (meaning treatment effects cannot clearly be attributed to the exercise), or if intervention groups receive identical therapeutic exercise interventions. Titles and abstracts of identified studies and subsequently full papers will be independently reviewed by two reviewers. A third reviewer will be consulted to resolve disagreements if necessary.

For peer review only - http://bmjopen.bmj.com/site/about/guidelines.xhtml

Table 1: Inclusion/ exclusion criteria

	Inclusion criteria	Exclusion criteria
**Population**	• Knee and/or hip pain in adults aged 45 years and over (mean age over 45 years)<br>• Knee and/ or hip OA diagnosed by x-ray<br>• Knee and/ or hip OA diagnosed according to clinical criteria<br>• Knee and/ or hip OA diagnosed by health care professional<br>• Self-reported knee and/ or hip OA<br>N.B: If population is mixed (e.g. OA and rheumatoid arthritis (RA), include if over 50% of participants have OA	• Knee and/ or hip pain attributable to conditions other than OA<br>• Non-musculoskeletal conditions<br>• Rheumatoid arthritis/ other defined inflammatory rheumatological problems<br>• Pre-operative patients (people on waiting lists for knee/hip surgery, including total joint replacement)<br>• Post-operative patients (immediately following knee/hip surgery, including total joint replacement)<br>• People with 'patellofemoral pain syndrome' (overall a different problem to 'OA')<br>• Animal based studies<br>• Studies of children
**Intervention**	• Any therapeutic exercise intervention (land or water based), regardless of content, duration, frequency, or intensity	• Non exercise interventions<br>• Advice only to exercise or increase physical activity, including within wider OA self-management programmes<br>• Exercise or physical activity that was not specifically applied to improve OA symptoms and function<br>• Exercise combined with other treatment modalities other than advice/ education/ self-

For peer review only - http://bmjopen.bmj.com/site/about/guidelines.xhtml

		management/ motivational techniques)<br><br>• Pre/post-operative exercise therapy, i.e. exercise immediately before, or following knee/hip surgery
**Comparator**	• Other forms of exercise (i.e. different type, duration, frequency or intensity of exercise if sufficiently different from the intervention arm), or no exercise control group (including usual care, waiting list, placebo, attention control, or no treatment)<br>• Sham treatment (e.g. sham ultrasound)	• If intervention groups receive identical therapeutic exercise interventions (i.e. no contrast existing between the intervention groups)<br><br>• If the comparator is a different intervention other than usual care (for example manual therapy, ultrasound, intra-articular injection, opioids, weight loss etc.), waiting list, placebo, attention control, or no treatment
**Outcome measure**	• Any self-reported measure of pain and/or physical function	• No measure of self-reported pain and/or physical function
**Study design**	• Randomised controlled trial<br><br>• Quasi-randomised controlled trial (where the method of allocation is known, but is not considered strictly random, e.g. alternation, date of birth, medical record number).	• Non-randomised controlled trial study design<br><br>• Other study designs e.g. surveys, observational studies, pre-post experiments (without a control group), qualitative studies<br><br>• Systematic reviews<br><br>• RCT protocols

For peer review only

For peer review only - http://bmjopen.bmj.com/site/about/guidelines.xhtml

Extraction of aggregate data

For each included trial, details on design, sample size, population characteristics (knee OA, hip OA or mixed), interventions (type, duration, exercise deliverer), comparator, candidate baseline variables (potential treatment moderators and mediators), and outcome assessment (type of outcome measure, length of follow-up) will be extracted and summarised into tables. Two reviewers will independently extract outcome data on self-report pain and/or physical function at time points nearest to 12 weeks, 6 months and 12 months post randomisation. Two reviewers will independently classify the exercise interventions of included trials, based on the following a priori defined characteristics:

Frequency of exercise: Number of exercise sessions per week.

Intensity of exercise: Low, moderate or high intensity (based on published information regarding target heart rate (<50% of maximum heart rate (MHR) = low intensity, 50–70% MHR = moderate intensity, >70–85% MHR = high intensity) or Metabolic Equivalent (MET) score (where heart rate information is unavailable) (MET score of <3 = low intensity, MET 3–6 = moderate intensity, MET >6 = high intensity [34])) [35]; low or high impact (categorised based on the likely amount of compressive load and whether both feet were intermittently off the ground. For example, cycling, swimming and walking = low impact; jogging, running and jumping = high impact) [35].

Type of exercise: Predominantly strengthening (e.g. quadriceps strengthening); predominantly general (aerobic) (e.g. walking, swimming); predominantly mind-body (e.g. yoga, tai-chi)[36]; mixed. As many trials are likely to include predominantly strengthening, we will classify subsets of predominantly non-weightbearing/ open

For peer review only

kinetic chain strengthening exercise versus predominantly weightbearing/ closed kinetic chain strengthening exercise.

Duration of exercise programme: Short (less than 6 weeks), or longer durations of up to 12 weeks, and over 12 weeks; total number of exercise sessions; booster sessions or no booster sessions.

Setting of exercise: Group, individual or mixed; supervised in clinic, completed at home or mixed; face-to-face, remote exercise instruction, or mixed.

Exercise deliverer: Healthcare professionals, exercise specialists, peer or lay-led, or mixed.

PHASE 2: COLLECTION, CHECKING AND STANDARDISING INDIVIDUAL PARTICIPANT DATA

In collaboration with, and following the procedures of, the OA Trial Bank, we will contact lead authors of identified trials to inform them about the study and invite them to share IPD. If the updated systematic review yields a large additional number of RCTs suitable for inclusion, and many authors are willing to share IPD, we may examine the likely power of the IPD meta-analysis accordingly to the trials promising their IPD using a simulation-based approach[37]. The collection, cleaning and harmonisation of IPD is often resource intensive[28,38], and therefore the power calculation will inform how much IPD is required in order to obtain sufficient power (e.g. 80%) to evaluate our key objectives. If IPD is ultimately sought from a subset of studies, this will be based on study quality, sample size and improvement to power, and will be independent of effect size to avoid selection bias[39].

For peer review only - http://bmjopen.bmj.com/site/about/guidelines.xhtml

Once a data sharing agreement is in situ, datasets will be accepted in any form, provided all data are anonymised and variables and categories are adequately labelled in English. To ensure accurate pooling of data, each dataset will be converted to a common format and variables re-named in a consistent manner.

Variables of interest

IPD to be obtained (where available) will include the following:

Baseline measures:

Socio-demographic factors: age, sex, co-morbidities, frailty, fatigue/ vitality, quality of life, body mass index, baseline physical activity level, previous lower limb injury, work status (working yes/no), manual versus non-manual work, previous physical load, family history of OA, socioeconomic status (education), social support, currently receiving other treatment, smoking status, motivation to exercise, previous knee injury/ trauma.

Psychological factors: anxiety/ depression, self-efficacy, outcome expectations.

Disease characteristics: pain location, pain elsewhere, pain severity, pain duration, central pain sensitisation, pain bothersomeness, physical function, stage of OA (early versus established OA[40]), radiographic joint structure, evidence of synovitis and bone marrow lesions from MRIs, patellofemoral OA damage.

Biomechanical factors: proprioception, static/ dynamic alignment, strength of hip and lower limb musculature, range of movement, leg length discrepancy, developmental hip abnormalities/ deformities.

For peer review only - http://bmjopen.bmj.com/site/about/guidelines.xhtml

For peer review only

Outcome measures: All self-report pain and physical function outcome data at time-points nearest to 12 weeks (short-term), 6 months (mid-term) and 1 year (long-term) post randomisation. If more than one measure of self-reported pain and physical function are reported, we will chose the highest in the hierarchy of outcome measures, as recommended by the Cochrane Musculoskeletal Review Group[41]. For pain these are: 1. pain overall; 2. pain on walking; 3. WOMAC pain subscale; 4. pain on activities other than walking; 5. WOMAC global scale; 6. Lequesne osteoarthritis index global score; 7. other algofunctional scale; 8. patient's global assessment; 9. physician's global assessment; 10. other outcome; 11. no continuous outcome reported. For physical function these are: 1. global disability score; 2. walking disability; 3. WOMAC disability subscore; 4. composite disability scores other than WOMAC; 5. disability other than walking; 6. WOMAC global scale; 7. Lequesne osteoarthritis index global score; 8. other algofunctional scale. Additionally, strength of hip and lower limb musculature, range of movement (total ROM, maximal flexion, maximal extension, extension deficit), and proprioception measures will be obtained during, and directly post-intervention.

Data quality assurance

We will evaluate the IPD from each trial to ensure the ranges of included variables are reasonable, and missing data will be checked against the original trial report. We will attempt to re-produce the results included in each initial trial publication, including baseline characteristics and self-reported pain and physical function at a time point nearest to 12 weeks, 6 months and 1 year post randomisation. Discrepancies or missing information will be discussed and clarified with original trial

For peer review only - http://bmjopen.bmj.com/site/about/guidelines.xhtml

authors. Where discrepancies cannot be explained the trial data will be excluded from the analysis. Individual trial datasets will be combined to form a new master dataset with a variable added to indicate the original trial.

Assessment of risk of bias

We will use the Cochrane Collaboration's tool for assessing risk of bias, based on publications of the included trials[42]. Two researchers will independently grade risk of bias (unclear, high, or low risk of bias) based on sequence generation, allocation concealment, blinding of outcome assessor, incomplete outcome data, and selective outcome reporting. Trial design, conduct and analysis methods will be clarified with Principal Investigators. Additionally, IPD will be directly checked for key potential biases, including whether baseline participant characteristics are balanced by arm. It will also be checked to ensure that data on all or as many randomised participants as possible are included, and any additional relevant outcome data from trials will be obtained.

PART 3: STATISTICAL ANALYSES

We will describe trial-level and participant-level characteristics (for baseline variables and outcomes) of included studies and examine if RCTs for which IPD are obtained are representative of the full set of existing RCTs by comparing key study characteristics, e.g. country of origin, sample size. All meta-analyses, apart from the mediation analyses (Objective 6 below), will use a two-stage approach, where each

For peer review only

For peer review only - http://bmjopen.bmj.com/site/about/guidelines.xhtml

trial is analysed separately in the first stage (which accounts for clustering of participants within trials) to produce effect estimates of interest, which are then synthesised in the second stage to produce summary meta-analysis results based on a random effects model (to account for between-trial heterogeneity)[26]. Analyses will be on an intention-to-treat principle and all summary estimates will be reported with 95% confidence intervals and *p*-values, with approaches such as Hartung-Knapp used to account for uncertainty of variance estimates[43,44]. Under a 'missing-at-random' assumption, individuals with partially missing outcome data will be included in analyses without imputation using a longitudinal data meta-analysis framework. If there is a considerable amount of missing baseline data (> 5% of patients have one or more missing values) for particular variables of interest (such as potential individual-level effect moderators) this will be handled using within-study multiple imputation[45] and Rubin's rule to estimate effects from imputed datasets[46]. All analyses will be carried out using Stata 14.1[47] or SAS 9.3[48].

Objective 1: For the meta-analysis to estimate an overall intervention effect (at 12 weeks, 6 months and 1 year) for self-reported pain and physical function, all available comparisons will be grouped into any exercise intervention versus a non-exercise control. Most outcomes will be continuous, so linear regression models will be fitted in the first stage. Longitudinal models will be used to account for the correlation between outcome values at the multiple time-points[49]. For each trial, the model will include baseline pain/physical function, treatment, time, and treatment by time interaction terms. The second stage requires a multivariate meta-analysis framework, which jointly syntheses the treatment effect estimates from the multiple time-points across trials[50]. Given the likely heterogeneity in the intervention effects

For peer review only - http://bmjopen.bmj.com/site/about/guidelines.xhtml

across trials (e.g. due to variability in patient characteristics), we will assume a multivariate random-effects meta-analysis model to estimate the summary results of interest using restricted maximum likelihood (REML) estimation. Heterogeneity in treatment effects across trials will be summarised by the estimated between-trial variance (τ^2) and multivariate I-squared statistics.

Objective 2: To determine which characteristics of exercise are associated with differences in overall effects, the meta-analysis approach in objective 1 will be repeated for particular subgroups of exercise interventions, including types, intensities, duration, setting or deliverer of exercise. To formally evaluate (albeit indirect) differences between exercise subgroups, meta-regression will be used. Trials comparing two different forms of exercise interventions will also be summarised, as these give direct (within-trial) information, which is preferable to indirect information. As appropriate, a sensitivity analysis will be performed to include direct and indirect evidence in one large (network) meta-analysis model.

Objectives 3 and 4: The IPD will be further analysed to examine treatment effect modification at the patient level, where individual patient characteristics are associated with differences in response to exercise. The models fitted in each trial will additionally include interaction terms between the intervention and patient-level covariates of interest to test effect modification. The interaction effect estimates at each time-point will then be pooled across trials using a multivariate random-effects meta-analysis. Covariates will be mean centred to aid the translation of results. Analyses of different exercise interventions (objective 4) will also be extended to examine treatment-covariate interactions in the same way, and identify subgroups of individuals likely to benefit most from specific types, intensities, duration, setting, and deliverer of exercise.

For peer review only

For peer review only - http://bmjopen.bmj.com/site/about/guidelines.xhtml

Objective 5: The analyses described for objectives 1 to 4 will be repeated in subgroups of people with only knee OA and only hip OA to examine whether the effect estimates differ by joint site.

Objective 6: As depicted in Figure 1 (panel A), in a mediation analysis an exposure can affect the outcome either through the mediator (E→M→O) or through other pathways (E→O). Using the counterfactual approach[51], the *total effect* of the exposure (exercise therapy) on the outcome (pain/physical function) through both pathways is determined. This effect can be decomposed into two components: the *direct effect* and the *indirect effect*. The *direct effect* refers to the causal pathway by which exercise therapy has an effect on pain/physical function not through the mediator. The *indirect effect* refers to the effect of exercise therapy that operates solely through the mediator under investigation. The counterfactual approach also allows for multiple mediators (Figure 1 panel B)[51]. Given the fact that in this approach the *total effect* can be decomposed into the *direct* and *indirect effects*, the percentage mediated by the mediator(s) can be calculated as an estimation of their importance.

In a one-stage approach, first the effect of the intervention (*a*) on the outcome (*Y*) will be determined, controlling for the mediator (*m*) under investigation and potential mediator-outcome confounders (*c*), using this model:

$$E[Y|a,m,c] = \theta_0 + \theta_1 a + \theta_2 m + \theta_3 am + \theta'_4 c$$

Next, the effect of the intervention on the mediator is determined, using this model:

$$E[M|a,c] = \beta_0 + \beta_1 a + \beta'_2 c$$

For peer review only - http://bmjopen.bmj.com/site/about/guidelines.xhtml

A single covariate will be added to both regression models to indicate each study, in order to adjust for possible residual confounding by study differences. Using these models, where θ_i and β_i are the regression coefficients, the natural direct effect (NDE) and natural indirect effect (NIE) are defined as:

$$NDE = \{\theta_1 + \theta_3(\beta_0+\beta_1 a+\beta'_2 c)\}$$

$$NIE = (\theta_2\beta_1 + \theta_3\beta_1 a)$$

and the total effect (TE) is equal to the sum of NDE and NIE[49]. Hence, the percentage mediated will be calculated by dividing NIE by TE and multiply this by 100%.

For knee OA, the mediating effect of upper leg muscle strength, extension deficits and proprioception will initially be analysed separately, using all data available for the IPD data meta-analysis. The effect of each potential mediator will then be evaluated adjusting for the other mediators in a multi-moderator model (Figure 1B). In the latter, a separate linear regression model will be calculated for each mediator[51] and the NIE, hence the percentage mediated, of each mediator can be calculated.

For hip OA, only the effect of muscle strength will be evaluated since this was the only factor indicated as a potential mediator in a systematic review[26]. In all analyses, the mediator will be defined as the absolute change from pre- to post-intervention. Therefore, the outcome measures that will be used will be those measured as close as possible to the end of the intervention period and to the measurement of the mediator(s) under investigation. All analyses will be run with and without stratification for type of exercise intervention.

For peer review only

For peer review only - http://bmjopen.bmj.com/site/about/guidelines.xhtml

Sensitivity analysis: Investigation of risk of bias

Effect estimates will be explored using data only from trials deemed to be at low risk of bias from: random sequence generation; allocation concealment; blinded outcome assessment; incomplete outcome data; and trials deemed to be at low risk of bias across all domains.

Unavailable IPD: For trials where IPD were not obtained, we will seek to extract suitable aggregate data from their trial publications and combine these with the IPD trials using suitable statistical methods, to determine whether conclusions remain the same[27]. This is only likely to be possible when examining overall effects, as subgroup differences (interactions) are rarely reported.

Investigation of small study effects: In meta-analyses of 10 trials or more, contour-enhanced funnel plots and tests for asymmetry will be used to investigate small trial effects and the potential for publication bias. Restriction to 10 trials is because there is low power to detect small trial effects with few studies[52].

ETHICS AND DISSEMINATION

Research ethical or governance approval is exempt for this study as no new data are being collected; [53,54]. Findings will be presented at national (UK) and international conferences, and submitted for publication in high-quality peer review journals. We will more broadly disseminate the results to physiotherapists, GPs, practice nurses, orthopaedic surgeons, patients and the general public both nationally and internationally by posting summaries on University websites, placing summaries in local health care settings and sending a summary to relevant groups and

For peer review only - http://bmjopen.bmj.com/site/about/guidelines.xhtml

organisations for wider dissemination e.g. Osteoarthritis Research Society International (OARSI), the European League Against Rheumatism (EULAR), the American College of Rheumatology (ACR) and Arthritis Research UK. Our PPIE working group will assist in developing plain English summaries and will jointly write articles that will be sent to newspapers, news websites, radio and other news media for wider dissemination.

TABLES AND FIGURES

Table 1: Inclusion/ exclusion criteria
Figure 1: Causal pathway of potential mediators (panel A: single mediator, panel B: multiple mediators)

AUTHORS' CONTRIBUTIONS

MAH, DLB, JR, DvdW, RDR, KD, NEF contributed to the initial conception of the study. MAH, DLB, JR, DvdW, RDR, KD, AL, ALE, HA, KB, JB, KLB, DB, LB, KC, RC, TC, MdR, MD,HPF, SH, RSH, MHR, MVH, CI, JK, IK, CM, SPM, DLP, NS, LAT, RT, CHT, MvM, CW and NEF made a substantial contribution to the design of the work. MAH, DLB and JR drafted the manuscript. MAH, DLB, JR, DvdW, RDR, KD, AL, ALE, HA, KB, JB, KLB, DB, LB, KC, RC, TC, MdR, MD,HPF, SH, RSH, MHR, MVH, CI, JK, IK, CM, SPM, DLP, NS, LAT, RT, CHT, MvM, CW and NEF revised the manuscript and approved the final submission. The OA trial bank steering committee peer reviewed and approved the study protocol. All authors agree to be accountable for all aspects of the work in ensuring that questions related to the accuracy or integrity of any part of the work are appropriately investigated and resolved. The guarantor of the review is NEF.

For peer review only

For peer review only - http://bmjopen.bmj.com/site/about/guidelines.xhtml

FUNDING STATEMENT

This work is supported by a Grant from the Chartered Society of Physiotherapy Charitable Trust (Grant number PRF/16/A07), and the National Institute for Health Research (NIHR) School of Primary Care Research (Grant number 531). MAH and DLB are supported by National Institute for Health Research (NIHR) School of Primary Care Research Fellowships. JR received partial funding from a grant of the Dutch Arthritis Foundation for their center of excellence 'osteoarthritis in primary care'. KB is funded by an Australian National Health and Medical Research Council Fellowship (#1058440). Musculoskeletal Statistics Unit, The Parker Institute, Bispebjerg and Frederiksberg Hospital (RC) is supported by a core grant from the Oak Foundation (OCAY-13-309). DvdW is a member of PROGRESS Medical Research Council Prognosis Research Strategy (PROGRESS) Partnership (G0902393/99558). KD is part-funded by a Knowledge Mobilisation Research Fellowship (KMRF-2014-03-002) from the NIHR and the NIHR Collaborations for Leadership in Applied Health Research and Care West Midlands. RSH is supported by an Australian Research Council Future Fellowship (FT130100175). NEF, a NIHR Senior Investigator, is supported through an NIHR Research Professorship (NIHR-RP-011-015). The funders did not influence the study design or the writing of this article. The views expressed in this publication are those of the authors and not necessarily those of the NHS, the NIHR or the Department of Health.

COMPETING INTERESTS

None to declare.

For peer review only - http://bmjopen.bmj.com/site/about/guidelines.xhtml

DATA SHARING STATEMENT

After this study has been completed, the individual participant data gathered will be deposited with the OA trial bank for the benefit of the wider OA community. Requests for future use of the data will be considered by the OA trial bank, and individual trial principal investigators, as applicable.

REFERENCES

1. National Institute for Health and Care Excellence. Osteoarthritis: care and management in adults. *NICE clinical guideline 177*. Royal College of Physicians, London, 2014.

2. Cross M, Smith E, Hoy D, et al. The global burden of hip and knee osteoarthritis: estimates from the global burden of disease 2010 study. *Ann Rheum Dis* 2014;73:1323-1330.

3. Lawrence RC, Felson DT, Helmick CG, et al. Estimates of the prevalence of arthritis and other rheumatic conditions in the United States. Part II. *Arthritis & Rheum* 2008;58:26-35.

4. Bennell KL, Hall M, Hinman RS. Osteoarthritis year in review 2015: rehabilitation and outcomes. *Osteoarthritis Cartilage* 2016;24:58-70.

5. Brosseau L, Rahman P, Toupin-April K, et al. A systematic critical appraisal for non-pharmacological management of osteoarthritis using the appraisal of guidelines research and evaluation II instrument. *PLoS One* 2014;9(1):e82986.

6. Nelson AE, Allen KD, Golightly YM, et al. A systematic review of recommendations and guidelines for the management of osteoarthritis: The Chronic

For peer review only - http://bmjopen.bmj.com/site/about/guidelines.xhtml

Osteoarthritis Management Initiative of the U.S. Bone and Joint Initiative. *Semin Arthritis Rheum* 2014;43:701-712.

7. Nicolson PJA, Bennell KL, Dobson FL, et al. Interventions to increase adherence to therapeutic exercise in older adults with low back pain and/or hip/knee osteoarthritis: a systematic review and meta-analysis. *Br J Sports Med* 2017;51:791-799.

8. Fransen M, McConnell S, Harmer AR, et al. Exercise for osteoarthritis of the knee. *Cochrane Database Syst Rev* 2015;1:CD004376. doi:10.1002/14651858.CD004376.

9. Fransen M, McConnell S, Hernandez-Molina G, et al. Exercise for osteoarthritis of the hip. *Cochrane Database Syst Rev* 2014;4:CD007912. doi:10.1002/14651858.CD007912.pub2.

10. Uthman OA, van der Windt DA, Jordan JL, et al. Exercise for lower limb osteoarthritis: systematic review incorporating trial sequential analysis and network meta-analysis. *BMJ* 2013;347:f5555.

11. Christensen R, Henriksen M, Leeds AR, et al. Effect of Weight Maintenance on Symptoms of Knee Osteoarthritis in Obese Patients: A Twelve-Month Randomized Controlled Trial. *Arthritis Care Res* 2015;67:640-650.

12. Foster NE, Thomas E, Barlas P, et al. Acupuncture as an adjunct to exercise based physiotherapy for osteoarthritis of the knee: randomised controlled trial. *BMJ* 2007;335:436.

13. Foster NE, Nicholls E, Holden MA, et al. Improving the effectiveness of exercise therapy for older adults with knee pain: a pragmatic randomised controlled trial (the beep trial). *Physiotherapy* 2015;e404-405.

For peer review only - http://bmjopen.bmj.com/site/about/guidelines.xhtml

14. Foster NE, Hill JC, O'Sullivan P, et al. Stratified models of care. *Best Pract Res Clin Rheumatol* 2013;27:649-661.

15. Hill JC, Whitehurst DG, Lewis M, et al. Comparison of stratified primary care management for low back pain with current best practice (STarT Back): a randomised controlled trial. *Lancet* 2011;378:1560-1571.

16. Kraemer HC, Wilson GT, Fairburn CG, et al. Mediators and moderators of treatment effects in randomized clinical trials. *Arch Gen Psychiatry* 2002;59:877-883.

17. Wright AA, Cook CE, Flynn TW, et al. Predictors of response to physical therapy intervention in patients with primary hip osteoarthritis. *Phys Ther* 2011;91:510-524.

18. French HP, Galvin R, Cusack T, et al. Predictors of short-term outcome to exercise and manual therapy for people with hip osteoarthritis. *Phys Ther* 2014;94:31-39.

19. Bennell KL, Dobson F, Roos EM, et al. Influence of biomechanical characteristics on pain and function outcomes from exercise in medial knee osteoarthritis and varus malalignment: exploratory analyses from a randomized controlled trial. *Arthritis Care Res* 2015;67:1281-1288.

20. Kudo M, Watanabe K, Otsubo H, et al. Analysis of effectiveness of therapeutic exercise for knee osteoarthritis and possible factors affecting outcome. *J Orthop Sci* 2013;18:932-939.

21. Knoop J, van der Leeden M, Roorda LD, et al. Knee joint stabilization therapy in patients with osteoarthritis of the knee and knee instability: subgroup analyses in a randomized, controlled trial. *J Rehabil Med* 2014;46:703-707.

For peer review only

22. Knoop J, Dekker J, van der Leeden M, et al. Is the severity of knee osteoarthritis on magnetic resonance imaging associated with outcome of exercise therapy? *Arthritis Care Res* 2014;66:63-68.

23. Foster NE, Thomas E, Hill JC, et al. The relationship between patient and practitioner expectations and preferences and clinical outcomes in a trial of exercise and acupuncture for knee osteoarthritis. *Eur J Pain* 2010;14:402-409.

24. Sun X, Ioannidis JP, Agoritsas T, et al. How to use a subgroup analysis: users' guide to the medical literature. *JAMA* 2014;311:405-11.

25. Juhl C, Christensen R, Roos EM, et al. Impact of exercise type and dose on pain and disability in knee osteoarthritis: a systematic review and meta-regression analysis of randomized controlled trials. *Arthritis Rheumatol* 2014;66(3):622-636.

26. Runhaar J, Luijsterburg P, Dekker J, et al. Identifying potential working mechanisms behind the positive effects of exercise therapy on pain and function in osteoarthritis; a systematic review. *Osteoarthritis Cartilage* 2015;23:1071-1082.

27. Riley RD, Lambert PC, Abo-Zaid G. Meta-analysis of individual participant data: rationale, conduct, and reporting. *BMJ* 2010;340:c221.

28. Tierney JF, Vale C, Riley R, et al. Individual Participant Data (IPD) Meta-analyses of Randomised Controlled Trials: Guidance on Their Use. *PLoS Med* 2015;12:e1001855.

29. Debray TP, Moons KG, van Valkenhoef G, et al. Get real in individual participant data (IPD) meta-analysis: a review of the methodology. *Res Synth Methods* 2015;6:293-309.

For peer review only - http://bmjopen.bmj.com/site/about/guidelines.xhtml

30. Stewart LA, Clarke M, Rovers M, et al. Preferred Reporting Items for a Systematic Review and Meta-analysis of Individual Participant Data: The PRISMA-IPD Statement. *JAMA* 2015;313:1657-1665.

31. INVOLVE. Public involvement in research: values and principles framework, INVOLVE: Eastleigh, 2015.

32. van Middelkoop M, Dziedzic KS, Doherty M, et al. Individual patient data meta-analysis of trials investigating the effectiveness of intra-articular glucocorticoid injections in patients with knee or hip osteoarthritis: an OA Trial Bank protocol for a systematic review. *Syst Rev* 2013;2:54.

33. van Middelkoop M, Arden NK, Atchia I, et al. The OA Trial Bank: meta-analysis of individual patient data from knee and hip osteoarthritis trials show that patients with severe pain exhibit greater benefit from intra-articular glucocorticoids. *Osteoarthritis Cartilage* 2016;S1063-4584(16)01002-5.

34. Ainsworth BE, Haskell WL, Herrmann SD, et al. Compendium of physical activities: a second update of codes and MET values. Med Sci Sports Exerc 2011;43(8):1575-1581.

35. Quicke JG, Foster NE, Thomas MJ, et al. Is long-term physical activity safe for older adults with knee pain?: a systematic review. Osteoarthritis Cartilage 2015;23(9):1445-56.

36. Brosseau L, Taki J, Desjardins B, et al. The Ottawa panel clinical practice guidelines for the management of knee osteoarthritis. Part one: introduction, and mind-body exercise programs. *Clin Rehabil* 2017;31:582-595.

For peer review only - http://bmjopen.bmj.com/site/about/guidelines.xhtml

37. Kontopantelis E, Springate DA, Parisi R et al. Simulation-Based Power Calculations for Mixed Effects Modeling: ipdpower in Stata. *Journal of Statistical Software* 2016;74:25.

38. Hróbjartsson A. Why did it take 19 months to retrieve clinical trial data from a non-profit organisation? *BMJ* 2013;347.

39. Ahmed I, Sutton AJ and Riley RD. Assessment of publication bias, selection bias and unavailable data in meta-analyses using individual participant data: a database survey. *BMJ* 2012;344:d7762.

40. Luyten FP, Denti M, Filardo G, et al. Definition and classification of early osteoarthritis of the knee. *Knee Surg Sports Traumatol Arthrosc* 2012;20:401-406.

41. Cochrane Musculoskeletal Group. URL: http://musculoskeletal.cochrane.org/proposed-outcomes (accessed 5th May 2017).

42. Higgins JPT, Green S (editors). *Cochrane Handbook for Systematic Reviews of Interventions* Version 5.1.0 [updated March 2011]. *The Cochrane Collaboration* 2011 www.handbook.cochrane.org (accessed 5th May 2017).

43. Hartung J, Knapp G. A refined method for the meta-analysis of controlled clinical trials with binary outcome. *Stat Med* 2001;20:3875-89.

44. Hartung J, Knapp G. On tests of the overall treatment effect in meta-analysis with normally distributed responses. *Stat Med* 2001;20:1771-82.

45. Quartagno M, Carpenter J.R. Multiple imputation for IPD meta-analysis: allowing for heterogeneity and studies with missing covariates. *Stats in Med* 2016;35:2938-54.

46. Carpenter JR, Kenward M. Multiple Imputation and its Application. Chichester: John Wiley & Sons 2013.

For peer review only - http://bmjopen.bmj.com/site/about/guidelines.xhtml

47. SAS Institute Inc. SAS/STAT_ 9.3 user's guide. Cary, NC: SAS Institute Inc, 2011.

48. StataCorp, Stata Statistical Software: Release 14. 2015, StataCorp LP: College Station, TX.

49. Jones AP, Riley RD, Williamson PR, et al. Meta-analysis of individual patient data versus aggregate data from longitudinal clinical trials. *Clin Trials* 2009;6:16-27.

50. Riley RD, Price MJ, Jackson D, et al. Multivariate meta-analysis using individual participant data. *Res Synth Methods* 2015;6:157-174.

51. VanderWeele TJ, Vansteelandt S. Mediation Analysis with Multiple Mediators. *Epidemiol Method* 2014;2:95-115.

52. Sterne JA, Sutton AJ, Ioannidis JP, et al. Recommendations for examining and interpreting funnel plot asymmetry in meta-analyses of randomised controlled trials. *BMJ* 2011;343:d4002.

53. Taichman DB, Backus J, Baethge C, et al. Sharing Clinical Trial Data: A Proposal From the International Committee of Medical Journal Editors. *JAMA* 2016;315:467-468.

54. Menikoff J. Letter from Jerry Menikoff, MD, JD. Director, Office for Human Research Protections, to ICMJE Secretairat. March 7, 2017. http://icmje.org/news-and-editorials/menikoff_icmje_questions_20170307.pdf (accessed 10th July 2017).

For peer review only

For peer review only - http://bmjopen.bmj.com/site/about/guidelines.xhtml

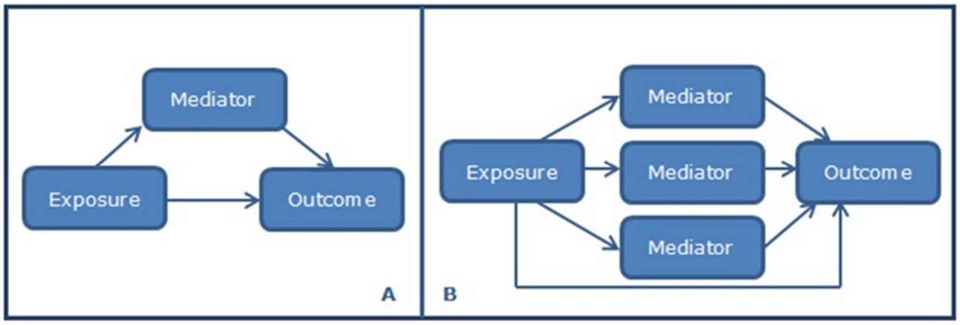

66x24mm (300 x 300 DPI)

For peer review only - http://bmjopen.bmj.com/site/about/guidelines.xhtml

Appendix 1: Full search strategies for Medline and Embase

Medline search	Embase search
1. randomized controlled trial.pt.<br>2. controlled clinical trial.pt.<br>3. randomized.ab.<br>4. placebo.ab.<br>5. drug therapy.fs.<br>6. randomly.ab.<br>7. trial.ab.<br>8. groups.ab.<br>9. therapy.fs.<br>10. 1 or 2 or 3 or 4 or 5 or 6 or 7 or 8 or 9<br>11. limit 10 to humans<br>12. exp Exercise Movement Techniques/ or exp Exercise/ or exp Exercise Therapy/<br>13. ((strength$ or isometric$ or isotonic$ or isokinetic$ or endurance or weight$) adj5 train$).ti,ab.<br>14. (resistance adj5 (exercis$ or train$)).ti,ab.<br>15. ((physical$ or motion$) adj5 (fit$ or therap$)).ti,ab.<br>16. (physical$ adj5 activ$).ti,ab.<br>17. physiotherap$.ti,ab.<br>18. kinesiotherap$.ti,ab.<br>19. rehab$.mp.<br>20. exp Physical Fitness/<br>21. (walk$ or jog$ or run$ or cycl$ or swim$ or treadmill$ or gym$ or bicycl$ or skat$ or row$).ti,ab.<br>22. exp Sports/<br>23. sport$.ti,ab.<br>24. exercise$.ti,ab.<br>25. aerobic$.ti,ab.<br>26. yoga.mp. or (tai adj1 (chi or ji)).ti,ab. [mp=title, original title, abstract, name of substance word, subject heading word, unique identifier]<br>27. danc$.ti,ab.<br>28. (aqua$ or water).ti,ab.<br>29. hydro$.ti,ab.<br>30. exp Hydrotherapy/<br>31. (stretch$ or flexib$ or balanc$ or propriocept$).ti,ab.<br>32. (circuit$ adj1 train$).ti,ab.<br>36. 12 or 13 or 14 or 15 or 16 or 17 or 18 or 19 or 20 or 21 or 22 or 23 or 24 or 25 or 26 or 27 or 28 or 29 or 30 or 31 or 32<br>37. exp Osteoarthritis/<br>38. osteoarthr$.ti,ab.<br>39. OA.ti,ab.<br>40. degenerative arthritis.mp.<br>41. arthrosis.mp.<br>42. 37 or 38 or 40 or 41<br>43. exp Knee Joint/ or exp Knee/<br>44. exp Hip Joint/ or exp Hip/<br>45. (knee$ or hip$).ti,ab.<br>46. 43 or 44 or 45<br>47. 42 and 46<br>48. ((knee$ or hip$) adj5 pain).ti,ab.<br>49. 47 or 48<br>50. 11 and 36 and 49	1. crossover procedure/<br>2. double-blind procedure/<br>3. randomized controlled trial/<br>4. single-blind procedure/<br>5. random$.ti,ab.<br>6. factorial$.ti,ab.<br>7. crossover$.ti,ab.<br>8. cross over$.ti,ab.<br>9. placebo$.ti,ab.<br>10. (doubl$ adj blind$).ti,ab.<br>11. (singl$ adj blind$).ti,ab.<br>12. assign$.ti,ab.<br>13. allocat$.ti,ab.<br>14. volunteer$.ti,ab.<br>15. 1 or 2 or 3 or 4 or 5 or 6 or 7 or 8 or 9 or 10 or 11 or 12 or 13 or 14<br>16. exp exercise/<br>17. exp kinesiotherapy/<br>18. fitness/<br>19. ((strength$ or isometric$ or isotonic$ or isokinetic$ or endurance or weight$) adj5 train$).ti,ab.<br>20. (resistance adj5 (exercis$ or train$)).ti,ab.<br>21. ((physical$ or motion$) adj5 (fit$ or therap$)).ti,ab.<br>22. (physical$ adj5 activ$).ti,ab.<br>23. physiotherap$.ti,ab.<br>24. kinesiotherap$.ti,ab.<br>25. rehab$.mp.<br>26. (walk$ or jog$ or run$ or cycl$ or swim$ or treadmill$ or gym$ or bicycl$ or skat$ or row$).ti,ab.<br>27. sport$.ti,ab.<br>28. exp SPORT/<br>29. exercise$.ti,ab.<br>30. aerobic$.ti,ab.<br>31. (yoga or (tai adj1 (chi or ji))).ti,ab.<br>32. danc$.ti,ab.<br>33. (aqua$ or water).ti,ab.<br>34. hydro$.ti,ab.<br>35. exp hydrotherapy/<br>36. (stretch$ or flexib$ or balanc$ or propriocept$).ti,ab.<br>37. (circuit$ adj1 train$).ti,ab.<br>38. or/16–37<br>39. exp OSTEOARTHRITIS/<br>40. osteoarthr$.ti,ab.<br>41. OA.ti,ab.<br>42. degenerative arthritis.mp.<br>43. arthrosis.mp.<br>44. 39 or 40 or 41 or 42 or 43<br>45. exp knee/<br>46. exp HIP/<br>47. (knee$ or hip$).ti,ab.<br>48. 45 or 46 or 47<br>49. 44 and 48<br>50. ((knee$ or hip$) adj5 pain).ti,ab.<br>51. 49 or 50<br>52. 15 and 38 and 51<br>53. limit 52 to human

For peer review only

For peer review only

PRISMA-P 2015 Checklist – STEER OA

This checklist has been adapted for use with protocol submissions to *Systematic Reviews* from Table 3 in Moher D et al: Preferred reporting items for systematic review and meta-analysis protocols (PRISMA-P) 2015 statement. *Systematic Reviews* 2015 **4**:1

Section/topic	#	Checklist item	Information reported		Line number(s)
			Yes	No	
**ADMINISTRATIVE INFORMATION**					
**Title**					
Identification	1a	Identify the report as a protocol of a systematic review	☒	☐	2
Update	1b	If the protocol is for an update of a previous systematic review, identify as such	☒	☐	2
**Registration**	2	If registered, provide the name of the registry (e.g., PROSPERO) and registration number in the Abstract	☒	☐	130
**Authors**					
Contact	3a	Provide name, institutional affiliation, and e-mail address of all protocol authors; provide physical mailing address of corresponding author	☒	☐	5-99
Contributions	3b	Describe contributions of protocol authors and identify the guarantor of the review	☒	☐	539-545
**Amendments**	4	If the protocol represents an amendment of a previously completed or published protocol, identify as such and list changes; otherwise, state plan for documenting important protocol amendments	☐	☒	
**Support**					
Sources	5a	Indicate sources of financial or other support for the review	☒	☐	577-596
Sponsor	5b	Provide name for the review funder and/or sponsor	☒	☐	578-580
Role of sponsor/funder	5c	Describe roles of funder(s), sponsor(s), and/or institution(s), if any, in developing the protocol	☒	☐	594-596
**INTRODUCTION**					
**Rationale**	6	Describe the rationale for the review in the context of what is already known	☒	☐	147-226
**Objectives**	7	Provide an explicit statement of the question(s) the review will address with reference to participants, interventions, comparators, and outcomes (PICO)	☒	☐	236-255

For peer review only

BioMed Central The Open Access Publisher

Section/topic	#	Checklist item	Information reported		Line number(s)
			Yes	No	
**METHODS**					
**Eligibility criteria**	8	Specify the study characteristics (e.g., PICO, study design, setting, time frame) and report characteristics (e.g., years considered, language, publication status) to be used as criteria for eligibility for the review	☒	☐	284-299, table 1
**Information sources**	9	Describe all intended information sources (e.g., electronic databases, contact with study authors, trial registers, or other grey literature sources) with planned dates of coverage	☒	☐	272-282
**Search strategy**	10	Present draft of search strategy to be used for at least one electronic database, including planned limits, such that it could be repeated	☒	☐	282, appendix 1
STUDY RECORDS					
Data management	11a	Describe the mechanism(s) that will be used to manage records and data throughout the review	☒	☐	319-351
Selection process	11b	State the process that will be used for selecting studies (e.g., two independent reviewers) through each phase of the review (i.e., screening, eligibility, and inclusion in meta-analysis)	☒	☐	295-298, 319-351
Data collection process	11c	Describe planned method of extracting data from reports (e.g., piloting forms, done independently, in duplicate), any processes for obtaining and confirming data from investigators	☒	☐	319-351
**Data items**	12	List and define all variables for which data will be sought (e.g., PICO items, funding sources), any pre-planned data assumptions and simplifications	☒	☐	319-351
**Outcomes and prioritization**	13	List and define all outcomes for which data will be sought, including prioritization of main and additional outcomes, with rationale	☒	☐	371-404
**Risk of bias in individual studies**	14	Describe anticipated methods for assessing risk of bias of individual studies, including whether this will be done at the outcome or study level, or both; state how this information will be used in data synthesis	☒	☐	406-427
DATA					
**Synthesis**	15a	Describe criteria under which study data will be quantitatively synthesized	☒	☐	429-541
	15b	If data are appropriate for quantitative synthesis, describe planned summary measures, methods of handling data, and methods of combining data from studies, including any planned exploration of consistency (e.g., I^2, Kendall's tau)	☒	☐	429-541
	15c	Describe any proposed additional analyses (e.g., sensitivity or subgroup analyses, meta-regression)	☒	☐	528-541
	15d	If quantitative synthesis is not appropriate, describe the type of summary planned	☐	☒	

For peer review only

BioMed Central The Open Access Publisher

Section/topic	#	Checklist item	Information reported: Yes	Information reported: No	Line number(s)
**Meta-bias(es)**	16	Specify any planned assessment of meta-bias(es) (e.g., publication bias across studies, selective reporting within studies)	☒	☐	429-541
**Confidence in cumulative evidence**	17	Describe how the strength of the body of evidence will be assessed (e.g., GRADE)	☐	☒	

For peer review only

BioMed Central
The Open Access Publisher
